# Comparative Analysis of Phenolic Acid Profiles, Antioxidant Capacity, and Antimicrobial Activity of Honeys from Different Botanical and Geographical Origins

**DOI:** 10.3390/ijms27177848

**Published:** 2026-09-02

**Authors:** Corina-Bianca Ioniță-Mîndrican, Antoanela Popescu, Magdalena Mititelu, Denisa-Elena Dumitrescu, Carolina Negrei, Iuliana Stoicescu, Violeta Popovici, Carmen Elena Lupu, Alina Maria Holban, Irinel Adriana Badea, Leontina Elena Filipiuc, Eliza Oprea

**Affiliations:** 1 Department of Toxicology, Faculty of Pharmacy, “Carol Davila” University of Medicine and Pharmacy, 020956 Bucharest, Romania; corina-bianca.ionita-mindrican@drd.umfcd.ro; 2 Department of Botany and Cosmetology, Faculty of Pharmacy, “Ovidius” University of Constanta, 900470 Constanta, Romania; antoanela.popescu@univ-ovidius.ro; 3 Department of Clinical Laboratory and Food Safety, Faculty of Pharmacy, “Carol Davila” University of Medicine and Pharmacy, 020956 Bucharest, Romania; magdalena.mititelu@umfcd.ro; 4 Department of Organic Chemistry, Faculty of Pharmacy, “Ovidius” University of Constanta, 900470 Constanta, Romania; 5 Advanced Research and Development Center for Experimental Medicine “Prof. Ostin C. Mungiu”-CEMEX, “Grigore T. Popa” University of Medicine and Pharmacy, 700115 Iasi, Romania; leontina.filipiuc@umfiasi.ro; 6 Department of Chemistry and Quality Control of Drugs, Faculty of Pharmacy, “Ovidius” University of Constanta, 900470 Constanta, Romania; iuliana.stoicescu@univ-ovidius.ro; 7 “Costin C. Kiriţescu” National Institute of Economic Research—Center for Mountain Economics (INCE-CEMONT) of Romanian Academy, 725700 Vatra-Dornei, Romania; violeta.popovici@ce-mont.ro; 8 Department of Mathematics and Informatics, Faculty of Pharmacy, “Ovidius” University of Constanta, 900001 Constanta, Romania; clupu@univ-ovidius.ro; 9 Department of Botany and Microbiology, Faculty of Biology, University of Bucharest, 060101 Bucharest, Romania; alina.m.holban@bio.unibuc.ro (A.M.H.); eliza.oprea@g.unibuc.ro (E.O.); 10 Research Institute of the University of Bucharest—ICUB, University of Bucharest, 050663 Bucharest, Romania; 11 Department of Analytical Chemistry and Physical Chemistry, Faculty of Chemistry, University of Bucharest, 050663 Bucharest, Romania; irinel.badea@chimie.unibuc.ro

**Keywords:** honey varieties, phenolic acid profile, bioactive compounds, antioxidant capacity, antimicrobial activity, microorganism anti-adherence on substrates

## Abstract

The composition of honey is complex and can vary depending on its botanical and geographical origin. This study evaluated the phenolic acid profile (PAP), antioxidant activity, and antimicrobial properties of honey samples from different geographical origins, such as Romania (nine types: multiflower, thyme, manna, hawthorn, black locust, linden, rapeseed, mint, and pasture), Malaysia (Tualang), New Zealand (Manuka), and Spain (chestnut). Twelve different varieties of honey were analyzed, one sample for each type, and all analyses were performed in triplicate. Phenolic acids were extracted by solid-phase extraction (SPE) using C18 cartridges, followed by high-performance liquid chromatography with diode-array detection (HPLC-DAD) analysis at 310 nm. Antioxidant activity was assessed using the 2,2′-diphenyl-1-picrylhydrazyl (DPPH) and 2,2′-azino-bis(3-ethylbenzothiazoline-6-sulfonic acid) (ABTS) assays. Antimicrobial activity was evaluated by agar diffusion and broth microdilution to determine the minimum inhibitory concentration (MIC), while microbial anti-adherence activity was assessed using the crystal violet staining assay to determine the minimum anti-adherence inhibitory concentration (MAIC). The tested microorganisms included the reference strains *Pseudomonas aeruginosa* ATCC 27853, *Escherichia coli* ATCC 25922, *Staphylococcus aureus* ATCC 25923, *Enterococcus faecalis* ATCC 29212, and *Candida albicans* ATCC 10231, as well as two *C. albicans* isolates (24114 and 5329). Several phenolic acids, including chlorogenic, caffeic, ferulic, *trans*-cinnamic, 3-O-methylgallic, *p*-coumaric, and syringic acids, were identified at varying concentrations across the analyzed honey samples. Rapeseed honey exhibited the highest overall concentration of phenolic acids. Among the honey samples analyzed, chestnut honey had the highest concentration of cinnamic acid, mint honey the highest concentration of ferulic acid, and multiflower honey the highest concentration of syringic acid. Tualang honey exhibited the highest antioxidant activity by both methods, reaching 2.103 mg TE/g honey for DPPH and 1.701 mg TE/g honey for ABTS. Tualang honey also exhibited the strongest antimicrobial activity against *C. albicans* and *P. aeruginosa*, whereas multifloral honey was the most active against *E. faecalis*. The analyzed honey extracts differed in their phenolic acid composition and biological activities, including antioxidant, antimicrobial, and anti-adherence effects of microorganisms on substrates. While associations were observed between selected phenolic acids and some of the evaluated biological activities, the overall biological properties of honey are more likely to arise from the combined contribution of multiple bioactive constituents than from individual compounds alone. The novelty of this study lies in the comparative assessment of the PAP obtained following SPE, and the biological activities of different types of honey from various botanical and geographical sources.

## 1. Introduction

The combination of honey’s antioxidant and antimicrobial properties in foods has attracted considerable interest due to its potential contribution to human health [1,2]. Honey contains several classes of bioactive compounds that may contribute to its antioxidant, antimicrobial, anti-inflammatory, and wound-healing properties [3,4,5,6]. It has been and remains a special natural food, with a prebiotic effect [7], that, through the multitude of bioactive compounds in its composition, possesses essential properties for health [8,9,10,11].

Honey contains over 180 compounds, including water, carbohydrates, enzymes, free amino acids, proteins, minerals, and vitamins, as well as other phytocompounds. The proportions of these compounds, as well as the floral source, climate, geographical area, storage conditions, and bee species, influence the taste and color of honey varieties [1,12,13].

Carbohydrates in honey contain different types of mono- and disaccharides. In the largest quantities and with the greatest contribution to the energy value are glucose and fructose. Other carbohydrates commonly found in honey include sucrose, maltose, turanose, isomaltose, nigerose, cellobiose, isopanose, and others, each present in varying proportions [1,14,15]. In the case of prolonged and improper storage of honey at high temperatures, the saccharides in its composition can lead to the formation of compounds that usually result from the Maillard reaction, including unwanted pentose (furfural) and hexose (5-hydroxymethylfurfural-HMF) compounds [16]. HMF is formed mainly through the acid-catalyzed dehydration of hexoses, particularly fructose, during prolonged storage or thermal processing [1,17].

In addition to the multitude of carbohydrates, there is also a small amount of protein in honey that can be found in the form of enzymes and amino acids. Proline is the main amino acid in the composition of honey (over 50%), an amino acid used as an indicator of honey maturity [1,18,19]. Besides botanical and geographical origin, honey composition may also be influenced by other biological and environmental factors, including the species or subspecies of honey bees and environmental conditions [20]. Studies comparing honeys of similar botanical and geographical origin produced by different *Apis* species or subspecies have reported differences in sugar and mineral profiles, and enzyme activities [21].

Depending on the floral origin, small amounts of vitamins (riboflavin, niacin, pantothenic acid, ascorbic acid) and minerals (magnesium, iron, copper, zinc, sodium, selenium) can be found in honey [22,23,24]. In general, the bioactive compounds present in honey can be classified into two classes: those belonging to the class with predominantly antibacterial properties and those belonging to the class with predominantly antioxidant properties [25,26,27,28]. Considering the influence of botanical and geographical origin on the composition of honey, attention has been given to phenolic acids and their relationship with the biological activities of honey.

Among the biological properties of honey, antimicrobial activity has attracted particular interest due to its potential applications in wound management and the growing concern over antimicrobial resistance [29]. For example, Manuka honey is known for its antibacterial activity supported in particular by the considerable amounts of methylglyoxal [30,31,32,33], but also possibly by the presence of compounds such as leptosperine [34]. The methylglyoxal present in Manuka honey has been shown to originate from dihydroxyacetone, which is present in the flowers of the Manuka tree (*Leptospermum scoparium*) [29,34]. Manuka honey is also characterized by a complex phytochemical composition associated with antioxidant activity [35].

An important role in the antioxidant activity of honey is played by polyphenols [36], compounds that protect the body from cellular damage by acting as a shield against oxidative stress. Polyphenols encompass a broad class of compounds normally found in plants. Their presence in honey varies according to the geographical region, but also the botanical source, the latter can represent a tool for authenticating the honey. The class of polyphenols includes phenolic acids and flavonoids [37,38,39].

Analysis of phenolic compounds is considered a means of studying the geographical and botanical origins of honey. Among the phenolic compounds reported as markers for honey are kaempferol (for rosemary honey), quercetin (for sunflower honey), hesperetin (for citrus honey) [40].

The most common phenolic compounds in honey are both those from the class of phenolic acids (gallic acid, ellagic acid, caffeic acid, syringic acid) and from the class of flavonoids (quercetin, kaempferol, luteolin, apigenin, pinocembrin) [39]. Quercetin and kaempferol, with remarkable bioactivities, are particularly important compounds due to their antioxidant, anti-inflammatory, and antimicrobial properties [41].

Among phenolic acids, ellagic acid is recognized for its antioxidant [42], anti-inflammatory [43], and antifungal properties [44].

Another compound found in many types of honey, which has recently come to the attention of researchers, is caffeic acid. It is a phenolic acid recognized for its antioxidant and anti-inflammatory properties, as well as for its antiproliferative, antihyperglycemic, neuroprotective, and antidepressant effects [45,46]. Caffeic acid also exhibits antibacterial and antiviral effects, with results demonstrated by the inhibition of strains of *S. aureus* [46].

Vanillin is a phenolic aldehyde identified in some varieties of honey. Recent research has shown that vanillin exerts a synergistic effect by modulating gentamicin’s activity against *S. aureus* and *E. coli*. Vanillin also demonstrated a synergistic effect in association with norfloxacin against *P. aeruginosa* [47]. Vanillin has also been shown to have stronger antioxidant activity than ascorbic acid and Trolox (6-hydroxy-2,5,7,8-tetramethylchroman-2-carboxylic acid) in the ORAC (oxygen radical absorbance capacity) and OxHLIA tests (oxidative hemolysis inhibition assay) [48].

The mechanism of the antioxidant activity of polyphenols brings together several ways, among which it is brought to attention: their ability as hydrogen donors, the ability to capture free radicals through phenolic groups, and the ability to chelate metal ions, preventing the generation of reactive oxygen species associated with these ions, so all these mechanisms contribute to the inhibition of oxidative processes at the cellular level [49,50,51,52].

In a previous study, the honey samples included in the present research were characterized through melissopalynological and physicochemical analyses. The melissopalynological analysis confirmed the botanical origin of each type of honey. It was observed that the highest percentages of dominant pollen were recorded for rapeseed honey (96.86% Brassicaceae), chestnut honey (94.17% Fagaceae), and linden honey (84.45% Malvaceae) [53]. Moreover, the physicochemical analysis highlighted the quality parameters of the honey samples. For all honey varieties analyzed, the moisture content was below the maximum allowed limit of 20%, indicating proper storage. The content of reducing sugars, sucrose level, and diastase activity were within accepted limits, confirming the freshness and authenticity of the samples. Regarding HMF determination, most samples were within the permitted limits, except for Tualang and pasture honey [53]. Four of the honey samples investigated in the present study (Manuka, Tualang, manna, and chestnut honey) were recently incorporated into topical formulations because of their promising biological properties. However, their phenolic profiles and biological activities have not yet been comprehensively compared with those of other honey varieties from different botanical and geographical origins [54]. Although numerous studies have investigated the biological properties of individual honey types, direct comparisons of phenolic composition, antioxidant capacity, and antimicrobial activity among honeys from different geographical origins remain limited. Therefore, the present study provides a comparative evaluation of twelve individual honey samples (one sample per honey type) originating from Romania, Malaysia, New Zealand, and Spain. To achieve more selective extraction of phenolic acids and reduce matrix interferences prior to HPLC-DAD analysis, solid-phase extraction (SPE) was employed [55].

This study’s novelty lies in the comparative evaluation of honey samples from different botanical and geographical origins, combining SPE-based profiling of phenolic acids with assessments of antioxidant, antibacterial, antifungal, and microbial anti-adherence activities. This approach allows analysis of the relationship between variations in phenolic acid composition and the biological activities of different types of honey within a single experimental framework.

## 2. Results

### 2.1. HPLC Analysis of Phenolic Acid Profile

The developed HPLC-DAD method allowed clear separation of the phenolic compounds. Linearity, detection limits, and retention times for each compound were evaluated (Table 1).

HPLC-DAD analysis of the extracts revealed the highest concentrations of chlorogenic acid in rapeseed honey (128.69 ± 0.45 μg/g honey) and chestnut honey (39.79 ± 0.18 μg/g). For rapeseed honey extract as well, the highest values of caffeic acid (33.25 ± 0.45 μg/g) and 3-O-methylgallic acid (206.29 ± 4.56 μg/g) were recorded. In most of the samples, *trans*-cinnamic acid was evident, showing the highest concentrations in chestnut honey extract (20.53 ± 0.52 μg/g), Manuka honey extract (19.78 ± 0.56 μg/g), and multiflower honey extract (18.02 ± 0.39 μg/g). Additionally, *p*-coumaric acid was detected in most of the analyzed extracts, with the highest values in hawthorn honey extract (6.48 ± 0.22 μg/g), followed by mint honey extract (5.71 ± 0.18 μg/g) and thyme honey extract (4.22 ± 0.14 μg/g). On the other hand, syringic acid was identified at low concentrations in most honey extracts, except for multiflower honey (26.74 ± 0.41 μg/g), while vanillin was detected exclusively in chestnut honey extract (0.15 ± 0.02 μg/g). Some phenolic compounds were also qualitatively identified in honey samples, such as kaempferol (P2, P3, P5, P11, and P12), luteolin (P2-P4 and P10-P12), rutoside (P2 and P11), as well as gallic acid and ellagic acid, which were present in all samples. Also, none of the samples contained *trans*-resveratrol, vanillic acid, caftaric acid, scutellarin, or isorhamnetin. Based on the analytical performance of the chromatographic method, only compounds for which reliable quantitative data were obtained are reported in Table 2.

Statistically significant differences between phenolic acid contents in different honey varieties, assessed by Welch’s ANOVA and the Games–Howell post hoc test, were recorded in Table 3.

### 2.2. Analysis of Antioxidant Capacity

Antioxidant capacity was evaluated using the DPPH and ABTS radical-scavenging methods. These activities were expressed as percentage antioxidant activity (AA%), while antioxidant capacity was also reported relative to Trolox as mg Trolox equivalents per gram of honey (mg TE/g honey) [56,57].

#### 2.2.1. DPPH Radical Scavenging Capacity

Thus, in the DPPH test, the lowest AA value was recorded for linden honey extract (11.466%), followed by mint honey extract (19.766%) and hawthorn honey extract (21.055%). The percentage of antioxidant activity was highest for Tualang honey (49.25%), followed by chestnut honey (40.08%) and rapeseed honey (39.52%) (Table 4).

#### 2.2.2. ABTS Radical Scavenging Capacity

The highest antioxidant activity was observed for Tualang honey extract (44.560%), and rapeseed honey extract (41.900%). Likewise, the lowest antioxidant activity was observed in linden honey extract (18.721%), hawthorn honey extract (21.090%), and black locust honey extract (21.779%) (Table 5).

Statistically significant differences between antioxidant activity (%) of different honey varieties, evaluated by both assays, assessed by Welch’s ANOVA and the Games–Howell post hoc test, were recorded in Table 6.

### 2.3. Evaluation of Antimicrobial Activity

**Figure 1:**
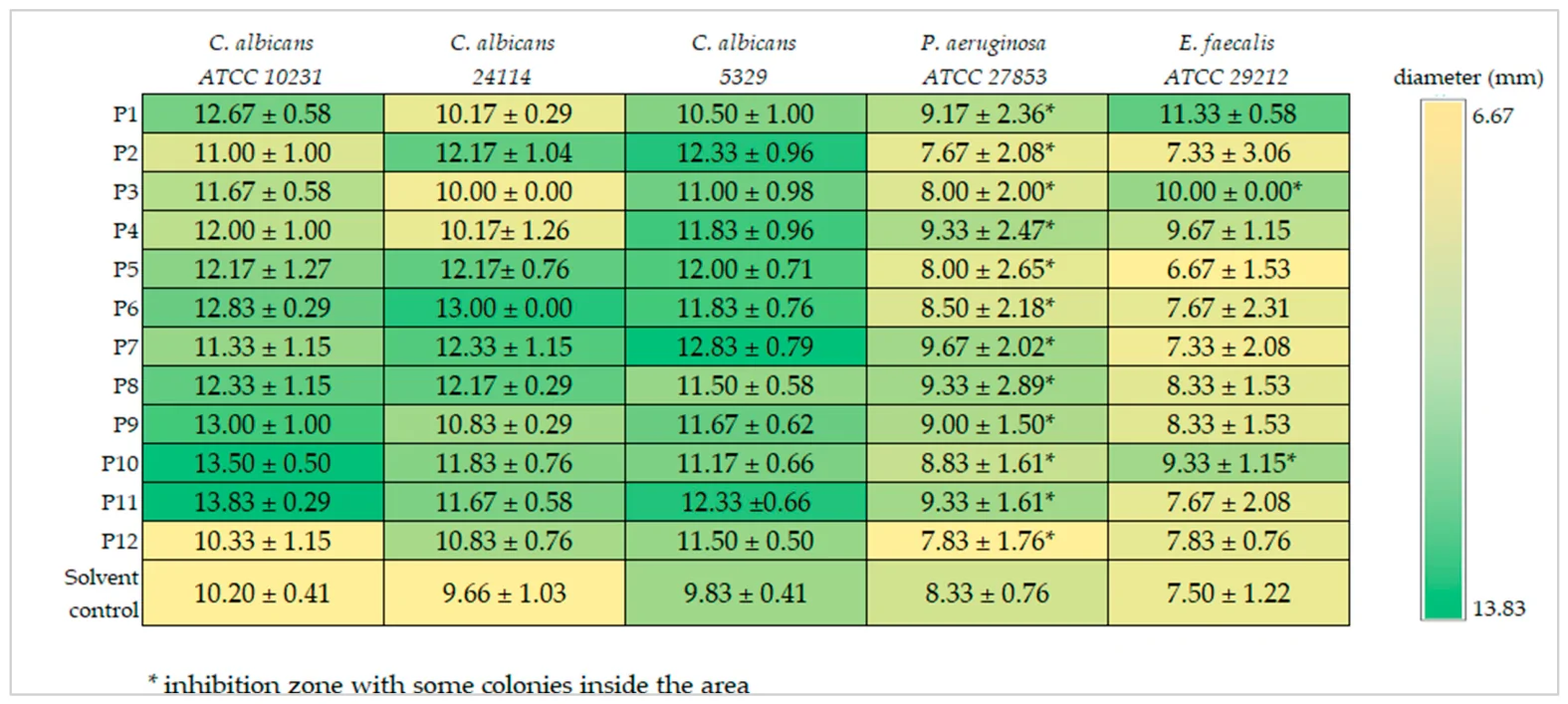
Heatmap of growth inhibition zone diameters (GIZD, mm) *of the honey extracts against the tested microbial strains*. P1: black locust honey; P2: multiflower honey; P3: chestnut honey; P4: thyme honey; P5: pasture honey; P6: manna honey; P7: mint honey; P8: hawthorn honey; P9: rapeseed honey; P10: linden honey; P11: Manuka honey; P12: Tualang honey.

The antimicrobial activity of the honey extracts was initially evaluated using the agar diffusion assay to measure growth inhibition zone diameters (GIZD). Overall, the honey extracts exhibited a more pronounced antifungal than antibacterial activity, as reflected by the larger inhibition zones observed against the *Candida albicans* strains. Although the methanol control also produced measurable GIZD (9.66–10.20 mm), all extracts and the control contained the same methanol concentration, allowing the inhibitory effects to be directly compared. Among the tested extracts, the Manuka honey extract produced the largest inhibition zone against *C. albicans* ATCC 10231 (13.83 ± 0.29 mm), followed by linden (13.50 ± 0.50 mm) and rapeseed (13.00 ± 1.00 mm) honey extracts. The manna honey extract showed the greatest activity against *C. albicans* 24114 (13.00 ± 0.00 mm). In contrast, the mint honey extract was the most active against *C. albicans* 5329 (12.83 ± 0.79 mm), followed by multifloral (12.33 ± 0.96 mm) and Manuka (12.33 ± 0.66 mm) honey extracts. In addition, considerably smaller GIZDs were observed against *P. aeruginosa* ATCC 27853 than against the *Candida albicans* strains. Moreover, bacterial colonies were frequently detected within the inhibition zones, indicating only partial inhibition of bacterial growth. A similar pattern was observed for *E. faecalis* ATCC 29212. Considering that the methanol control also produced measurable inhibition zones, the agar diffusion assay suggested that the additional inhibitory effect of the honey extracts was more evident against *Candida* spp. than that of the tested bacterial strains (Figure 1).

The differences between these variables, assessed using Welch’s ANOVA and the Games–Howell post hoc test, are reported in Table 7.

**Figure 2:**
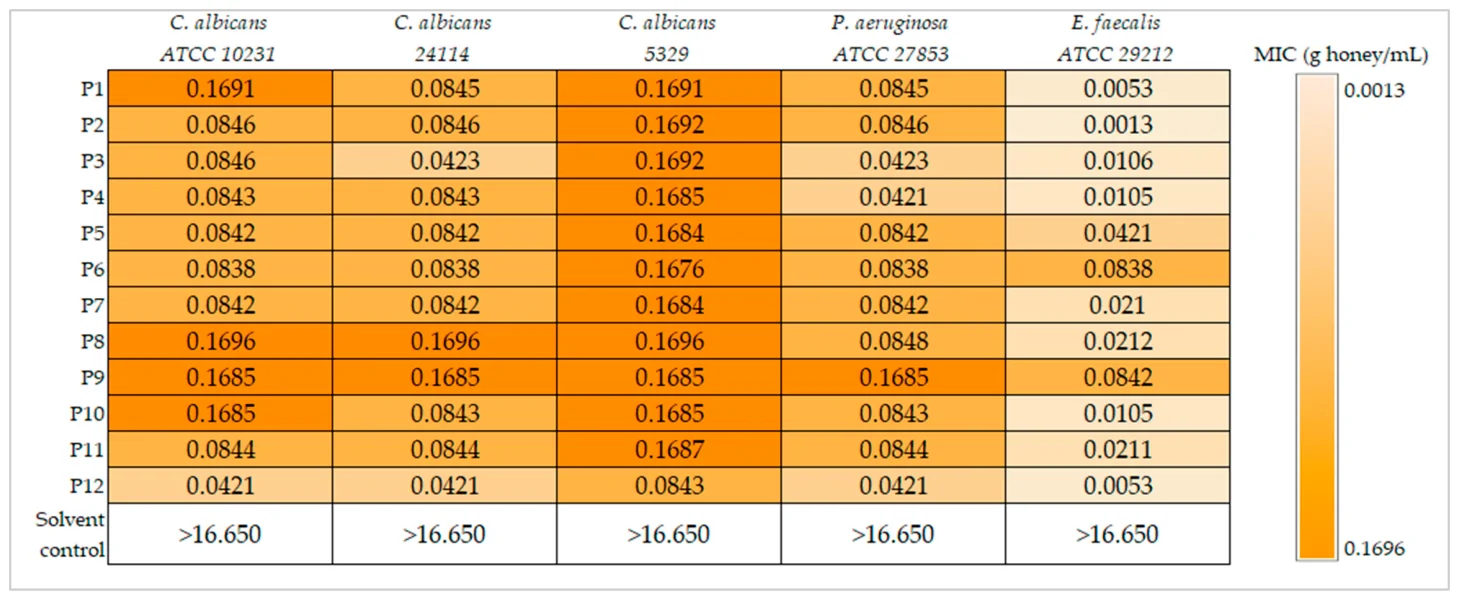
Heatmap of minimum Inhibitory Concentration (MIC), expressed as g honey/mL. P1: black locust honey; P2: multiflower honey; P3: chestnut honey; P4: thyme honey; P5: pasture honey; P6: manna honey; P7: mint honey; P8: hawthorn honey; P9: rapeseed honey; P10: linden honey; P11: Manuka honey; P12: Tualang honey.

The MIC values confirmed the antimicrobial activity of the analyzed honey extracts, although the results varied depending on both the extract and the tested microorganism. For the *Candida albicans* strains, MIC values ranged from 0.0421 to 0.1696 g honey/mL. The Tualang honey extract exhibited the lowest MIC against *C. albicans* ATCC 10231 and *C. albicans* 24114 (0.0421 g honey/mL). In comparison, it also showed the highest antifungal activity against *C. albicans* 5329 (0.0843 g honey/mL). Among the bacterial strains, the lowest MIC values were generally recorded against *E. faecalis* ATCC 29212, with the multiflower honey extract exhibiting the greatest activity (0.0013 g honey/mL). Against *P. aeruginosa* ATCC 27853, the Tualang and thyme honey extracts showed the lowest MIC values (Figure 2).

### 2.4. Semiquantitative Assessment of Microbial Adherence to an Inert Substrate

**Figure 3:**
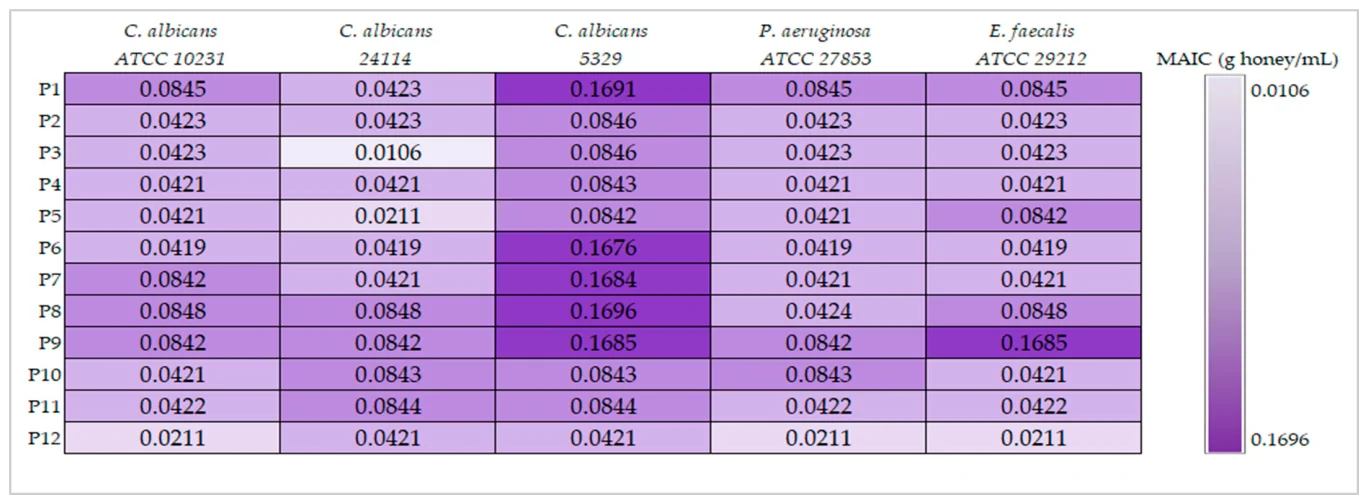
Heatmap of Minimum Adherence Inhibitory Concentration (MAIC), expressed as g honey/mL. P1: black locust honey; P2: multiflower honey; P3: chestnut honey; P4: thyme honey; P5: pasture honey; P6: Manna honey; P7: mint honey; P8: hawthorn honey; P9: rapeseed honey; P10: linden honey; P11: Manuka honey; P12: Tualang honey.

The MAIC values varied according to both the honey extract and the tested microorganism. Among the analyzed samples, the Tualang honey extract demonstrated the strongest anti-adherence activity, exhibiting the lowest MAIC values against four of the five tested microorganisms. Only *C. albicans* 24114 showed greater susceptibility to the chestnut honey extract, which exhibited the lowest MAIC value (0.0106 g/mL) (Figure 3).

**Figure 4:**
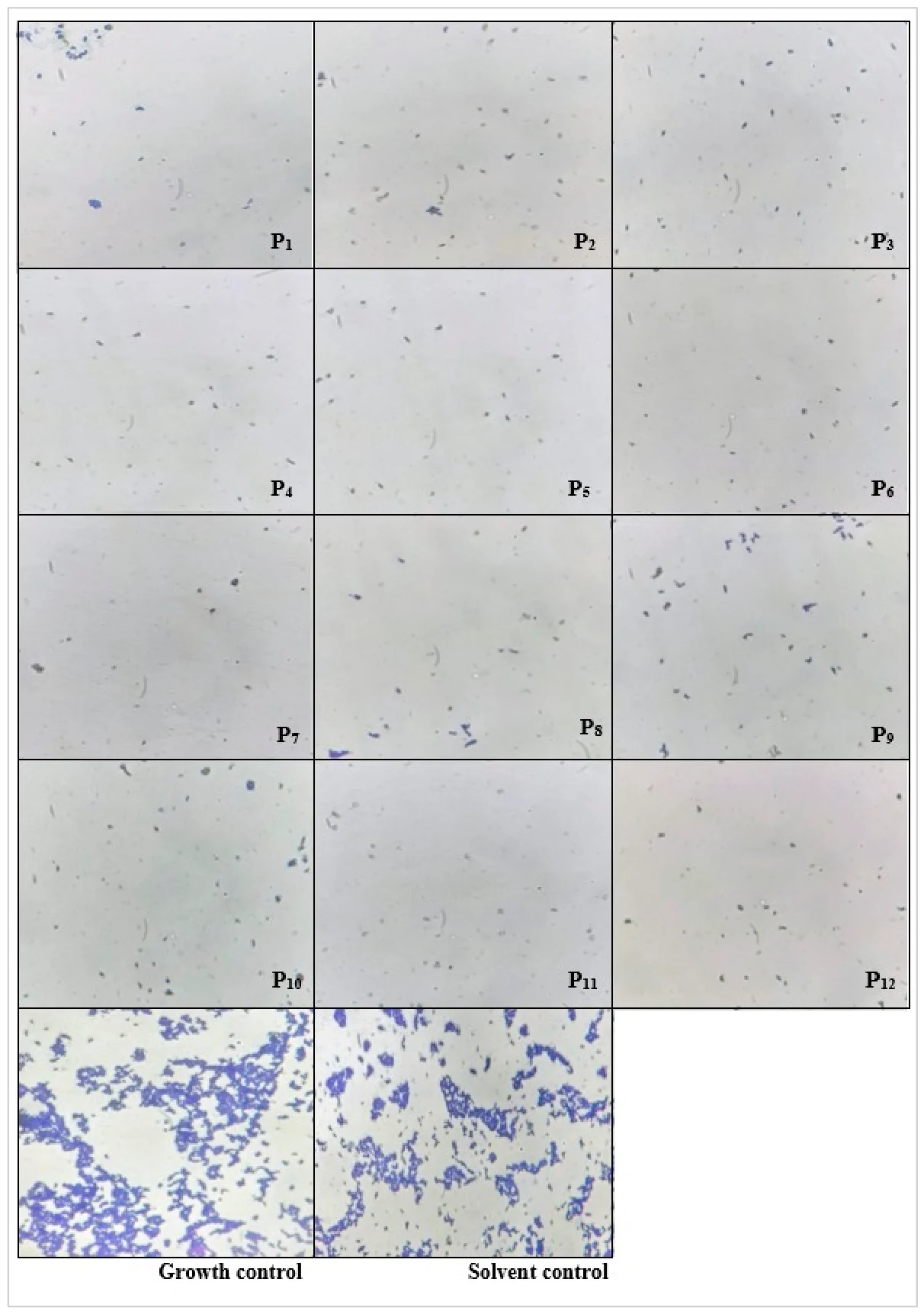
Optical microscopy images of microbial adherence in *C. albicans* 5329 in the presence of the honey extracts (inverted microscope, 400× magnification). P1: black locust honey; P2: multiflower honey; P3: chestnut honey; P4: thyme honey; P5: pasture honey; P6: manna honey; P7: mint honey; P8: hawthorn honey; P9: rapeseed honey; P10: linden honey; P11: Manuka honey; P12: Tualang honey.

Microscopic examination confirmed the anti-adherence activity of the analyzed honey extracts. Compared with the growth control, all extracts reduced the number of adherent cells in *C. albicans* ATCC 10231 (Figure S1), *C. albicans* 24114 (Figure S2), and *C. albicans* 5329 (Figure 4), whereas the solvent control had no appreciable effect.

Although *E. coli* and *S. aureus* were included among the tested microorganisms, no growth inhibition zones were detected for either strain under the conditions of this study. Consequently, MAIC values were not determined for these strains.

### 2.5. Data Analysis

The correlations between phenolic acids and antioxidant activity are displayed in Table 8.

*p*-Coumaric acid shows a moderate negative correlation with antioxidant activity determined through both methods (*r* = –0.5849, *r* = −0.7188, *p* < 0.05). Both antioxidant activities exhibit a strong intercorrelation (*r* = 0.9327, *p* < 0.001) (Table 8).

**Figure 5:**
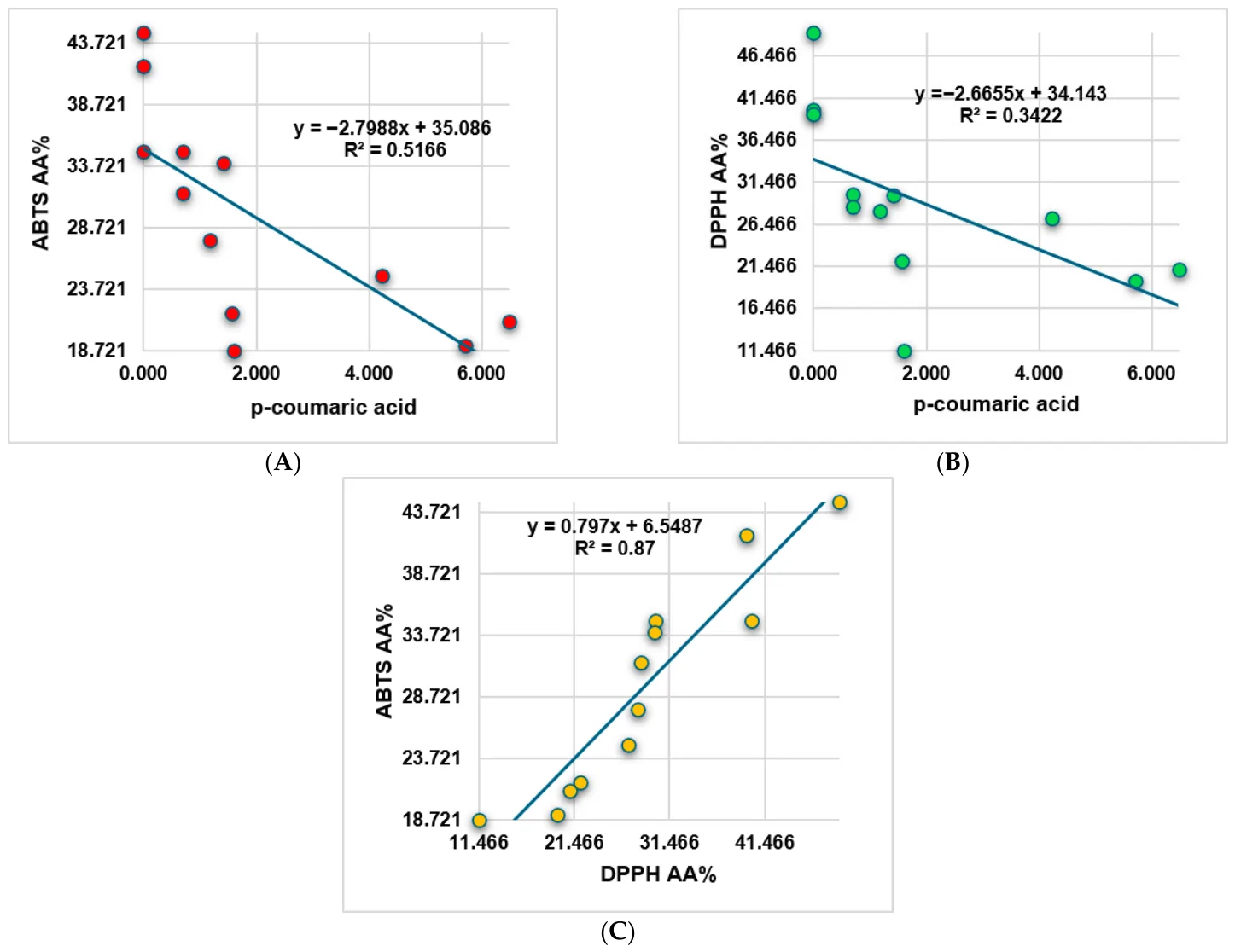
Scatterplot regression models of statistically significant correlations between antioxidant activity and *p*-coumaric acid (µg/g) (**A**,**B**): ABTS AA% (**A**) and DPPH AA% (**B**); covariance of ABTS AA% and DPPH AA% (**C**). R^2^ = coefficient of determination.

Only 51% of the variation in the ABTS AA% and only 34% of the variation in DPPH AA% are explained by the linear relationship with the *p*-coumaric acid content (Figure 5A,B). Moreover, 87% of the ABTS AA% variance is evidenced by its relationship with DPPH AA% (Figure 5C).

Chlorogenic acid, caffeic acid and 3-O-methylgallic acid exhibited a moderate positive correlation with antibacterial activity against *P. aeruginosa* ATCC 27853 (*r* > 0.70, *p* < 0.05) (Table 9).

**Figure 6:**
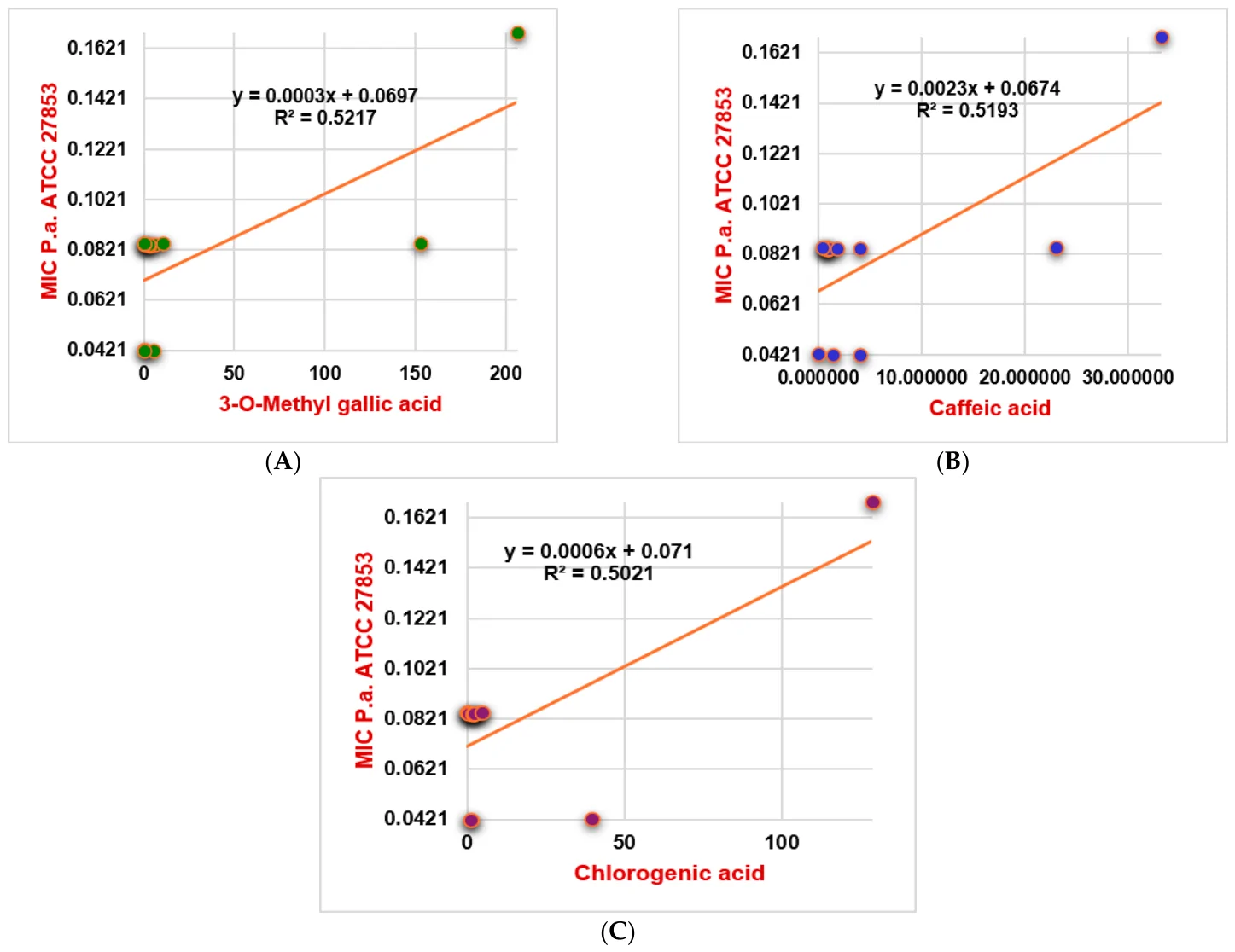
Scatter plots of the inhibitory activity of *P. aeruginosa* ATCC 27853 strain growth (expressed as MIC, g/mL) correlated with phenolic constituents (µg/g) of honey varieties: 3-O-methylgallic acid (**A**), caffeic acid (**B**), chlorogenic acid (**C**); R^2^ = coefficient of determination; P.a. = *P. aeruginosa;* MIC = minimum inhibitory concentration (g/mL).

Over 50% of the variation in MIC against *P. aeruginosa* 27853 can be explained by a linear relationship with 3-O-methylgallic acid, caffeic acid, and chlorogenic acid contents in honey varieties (Figure 6A–C).

These associations cannot be interpreted as evidence of enhanced antimicrobial activity with increasing concentrations of these phenolic acids, as the higher MIC values observed in our experiments indicate only modest inhibition of microbial growth.

High concentrations of phenolic constituents can display surprisingly low or negligible direct anti-*Candida* effects due to structural specificity, rapid metabolic transformation, and matrix binding. Simple phenolic acids often lack the specific functional groups required to disrupt fungal cell membranes or inhibit key enzymes [58]. They can also bind nonspecifically to proteins or media components (such as in complex extracts or food matrices), neutralizing their free availability to interact with *Candida* cells [59].

Our findings are consistent with scientific literature, which indicates that natural products with notable antioxidant potential simultaneously neutralize free radicals and disrupt fungal cell stability [60].

Several phenolic compounds show low direct antibacterial growth inhibition against *P. aeruginosa* because of the bacterium’s tough outer membrane, active efflux pumps, and the tendency of these plant compounds to act as anti-virulence agents rather than direct cell killers [61]. Various phenolic constituents can also show a low antibacterial effect against *E. faecalis* due to structural barriers, such as sugar attachments, strong biofilm protection, or low membrane affinity [61].

Most phenolic acids show positive correlations with the inhibition of microbial adherence to an inert substrate (Table 10). Significant positive correlations were observed between chlorogenic acid, caffeic acid, and 3-O-methylgallic acid and MAIC values against E. faecalis ATCC 29212 (*r* > 0.60, *p* < 0.05) (Table 10). Ferulic acid shows a moderate correlation with MAIC against *C. albicans* ATCC 10231 and 5329 (*r* > 0.60, *p* < 0.05). All negative correlations are low and not statistically significant (*p* > 0.05; Table 10).

**Figure 7:**
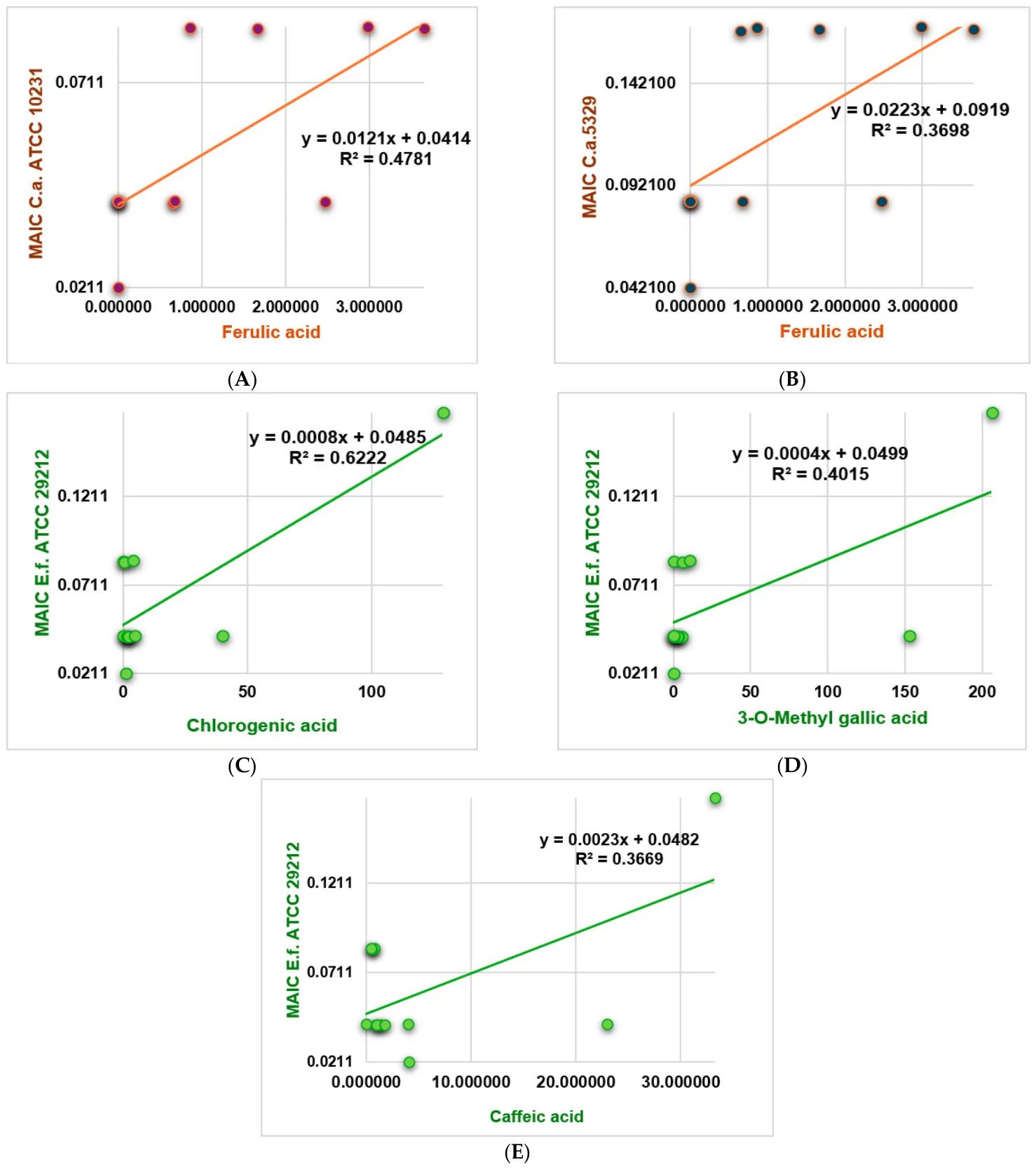
Scatter plots of the inhibitory activity of microbial adherence to an inert substrate (MAIC, g/mL) correlated with phenolic constituents (µg/g) of honey varieties: Inhibitory activity of *C. albicans* adherence (**A**,**B**); Inhibitory activity of *E. faecalis* adherence (**C**–**E**); R^2^ = coefficient of determination; C.a. = *C. albicans*; E.f. = *E. faecalis*.

Furthermore, only 47.81% of the variation in the MAIC against *C. albicans* ATCC 10231 and only 36.98% of the variation in the MAIC against *C. albicans* 5329 are explained by a linear relationship with ferulic acid content (Figure 7A,B). Similarly, MAIC against *E. faecalis* ATCC 29212 shows only 62% variation attributable to chlorogenic acid, 40% to 3-O-methylgallic acid, and 36% to caffeic acid (Figure 7C–E).

We also investigated potential correlations among other previously quantified constituents (reducing sugars, hydroxymethyl furfural), diastase activity in the tested honey varieties [53], and antioxidant and antibacterial properties (Table 11).

HMF shows a strong negative correlation with MIC against *C. albicans* 5329 (*r* = −0.8258, *p* < 0.05) and a moderate negative correlation with MIC against *C. albicans* 10231 (*r* = −0.5890, *p* < 0.05) (Table 11). HMF also reports a moderate positive correlation with antioxidant activity determined by both methods (*r* = 0.5, *p* > 0.05) (Table 11). On the other hand, antioxidant activity determined by the DPPH method (DPPH AA%) has a moderate negative correlation with the MIC against *C. albicans* 5329 *(r* = −0.6248, *p* < 0.05) (Table 11).

Our results align with literature data because it is known that HMF exhibits antimicrobial properties, including antioxidant, antifungal, and antibacterial activities, and serves as a precursor or active component in various pharmacological and food-related contexts [62,63]. In addition, previous research suggests that the phenolic constituents are associated with nearly all of the bioactivities of honey [1], and their antimicrobial activity varies directly with antioxidant potential.

In our study, only 12 honey varieties were analyzed; therefore, advanced multiple regression analyses were conducted to examine the relationship between a continuous dependent variable and two or more independent predictor variables. All detailed data are provided in the Supplementary Materials. Overall, the absence of statistically significant results in both residual and predictive models, R-square values < 90%, VIF values > 5, and significant autocorrelation between residuals suggests that these correlations should be interpreted with caution; the antioxidant and antimicrobial effects of honey likely arise from a combination of bioactive compounds rather than a single phenolic constituent.

## 3. Discussion

The present study is a comprehensive investigation of the phytochemical profiles and bioactivities of 12 honey varieties from different botanical and geographical origins. The analysis of phenolic compounds in the honey samples reveals significant differences primarily influenced by the source of origin (floral nectar or manna) and the geographical conditions of production. These findings highlight the diverse bioactive profiles of each honey variety and their potential therapeutic applications.

The honey samples exhibited substantial differences in their phenolic profiles, confirming the strong influence of botanical and geographical origin on honey composition. Rapeseed honey contained the highest concentrations of chlorogenic acid, caffeic acid, and 3-O-methylgallic acid. The relatively high concentrations found in the rapeseed honey sample should be interpreted with caution, as methodological factors, including the extraction procedure, can affect the phenolic acid content of honey. In contrast, chestnut honey exhibited elevated cinnamic acid concentrations and was the only sample in which vanillin was detected. Multifloral honey showed the highest syringic acid content. Similar differences among honey varieties have been reported previously and reflect differences in floral sources and environmental conditions [64,65].

The antioxidant activity of the analyzed honeys varied among varieties, with Tualang honey showing the highest antioxidant capacity in both the DPPH and ABTS methods. Although these assays involve different radical species and reaction mechanisms, Tualang honey’s consistent performance in both methods indicates a comparatively high overall radical-scavenging capacity. While the DPPH assay primarily reflects the ability of antioxidants to donate hydrogen atoms in an organic medium, the ABTS assay applies to both hydrophilic and lipophilic antioxidants. It is generally considered more responsive to a broader range of antioxidant compounds. Consequently, different honey samples may exhibit different rankings depending on their phenolic composition and the relative contribution of individual antioxidant constituents [66,67,68].

The higher antioxidant activity of Tualang honey, despite its lower concentrations of the quantified phenolic acids, suggests that the honey antioxidant capacity cannot be explained only by the individual phenolic constituents determined in the present study.

Therefore, the antioxidant capacity of honeys likely arises from the combined contributions of multiple bioactive compounds, including unquantified phenolic compounds, enzymes, vitamins, proteins, organic acids, and Maillard reaction products, all of which are naturally present in most types of honey. Furthermore, the distinct reaction mechanisms of the DPPH and ABTS assays may contribute to the observed differences in antioxidant ranking among the honey samples analyzed. Similar observations have been reported in previous studies, which emphasize that honey antioxidant activity depends not only on the phenolic compounds concentration but also on their qualitative composition and interactions with other constituents of the honey matrix [69,70].

Pearson correlation analysis revealed a strong positive correlation between the antioxidant activities determined by the DPPH and ABTS methods (*r* = 0.9327, *p* < 0.001), indicating a high degree of agreement between these assays. Among the quantified phenolic acids, *p*-coumaric acid showed a moderate negative correlation with antioxidant activity measured by both DPPH and ABTS (*p* < 0.05). No other significant correlations were observed between individual phenolic acids and antioxidant activity.

The antimicrobial activity of the honey samples varied considerably depending on honey variety and the tested microorganisms. Overall, the *C. albicans* strains tested were more susceptible to the honey extracts than the bacterial strains evaluated, as reflected by the MIC and MAIC values. The notable antifungal effect observed in our study may reflect differences in cellular organization and stress responses between yeasts and bacteria, as well as the simultaneous action of various bioactive constituents of honey samples. Similar observations have been reported previously for *Candida* spp. and other clinically relevant yeasts [71,72]. The inhibitory activity against *C. albicans* strains may be of particular interest, given the clinical relevance of *Candida* infections and the growing interest in honey-based products in antifungal complementary therapy. Previous studies have demonstrated in vitro activity of medical-grade honey formulations against some clinical *C. albicans* isolates, and their potential application in recurrent vulvovaginal candidiasis is receiving increasing attention [73,74]. Generally, the antimicrobial activity of honey is multifactorial, involving low pH, high osmolarity, hydrogen peroxide, bee-derived peptides (such as defensin-1), and other constituents; the relative contribution of these factors differs among honey varieties [75,76]. Nevertheless, these findings are limited to in vitro antimicrobial screening and cannot be directly extrapolated to clinical efficacy. Further studies using a broader panel of clinical and antifungal-resistant isolates, along with cytotoxicity assessments, mechanistic investigations, and in vivo models, are required to determine whether the observed antifungal activity is clinically relevant.

An additional strength of the present study is the simultaneous evaluation of both antimicrobial and anti-adherence activities of a diverse panel of honey varieties. While most previous studies have focused primarily on MIC determination, assessing microbial adherence provides complementary information on the ability of honey extracts to interfere with the initial stages of microbial surface colonization. This aspect is particularly relevant for opportunistic pathogens such as *C. albicans*, whose pathogenicity largely depends on adhesion to host tissues and abiotic substrates.

In the present study, all honey extracts reduced microbial adherence to different extents, with Tualang honey exhibiting the lowest MAIC values against most of the tested microorganisms. These findings suggest that certain honey varieties may not only inhibit microbial growth but also influence the establishment of surface-associated microbial communities, indicating their ability to interfere with microbial adherence to the tested inert substrate under the experimental conditions used.

Microscopic examination supported the quantitative MAIC results by demonstrating a visible reduction in the number of adherent *Candida* cells following treatment with the honey extracts. The qualitative agreement between microscopic observations and MAIC values strengthens the evidence that the investigated honeys interfere with the early stages of microbial surface colonization rather than merely reducing planktonic growth.

The combined evaluation of MIC, MAIC, and microscopic observations provides a more comprehensive assessment of the antimicrobial potential of honey than growth inhibition alone. This integrated approach highlights the ability of selected honey varieties, particularly Tualang honey, to interfere with the earliest stages of microbial colonization, supporting their in vitro anti-adherence activity.

In contrast, multifloral honey showed the highest inhibitory activity against *Enterococcus faecalis*. Numerous floral sources led to a highly variable chemical composition, which may result in enhanced activity against specific microorganisms [77]. Accordingly, the comparatively strong antimicrobial activity observed for Tualang honey and the activity of multifloral honey against *E. faecalis* cannot be attributed, based on the present data, to higher concentrations of a specific phenolic acid.

*P. aeruginosa* was the most resistant Gram-negative bacterium, requiring higher extract concentrations for growth inhibition than the fungal strains. This observation agrees with previous studies and is generally attributed to the intrinsic resistance of *P. aeruginosa*, which possesses a low-permeability outer membrane, efficient efflux systems, and the ability to tolerate a wide range of antimicrobial agents [78].

Cinnamic acid, reported to exhibit antimicrobial and anti-biofilm activity, was found in relatively high concentrations in the chestnut (P3) and Manuka honey extracts (P11) [79]. However, the present study does not establish a causal relationship between cinnamic acid concentration and the antimicrobial activity of these honey samples.

Mint honey had the highest concentration of ferulic acid, which has previously been reported to have antioxidant and antimicrobial activities. However, the present data do not allow the biological activity of mint honey to be attributed to ferulic acid, as the observed effects may reflect the honey matrix’s overall composition [80].

Rapeseed honey extract (P9) contained the highest concentrations of chlorogenic and caffeic acids and showed high antioxidant activity. Although both compounds have well-documented radical- scavenging properties [81,82], the present data cannot establish their individual contributions to the antioxidant activity of rapeseed honey. On the other hand, the antimicrobial activity of chlorogenic acid and caffeic acid may be due to disruption of bacterial membrane integrity. Therefore, by losing intracellular constituents, such as nucleotides, bacterial development is compromised [83,84].

Pearson correlation analysis evidenced that MIC values against several microbial species vary similarly among the analyzed honey extracts, suggesting partially shared susceptibility patterns. Interestingly, chlorogenic acid, caffeic acid, and 3-O-methylgallic acid showed significant positive correlations with MIC values against the tested *P. aeruginosa* strains; these indicate that higher concentrations of these compounds were associated with higher, rather than lower, MIC values. This finding suggests that the antibacterial activity of the honey extracts against *P. aeruginosa* cannot be directly explained by increasing concentrations of these individual phenolic acids. Thus, the antimicrobial activity results are more likely to reflect the combined contribution and interactions of multiple constituents within the complex honey matrix than the concentration of any quantified phenolic acid.

The complex design of the present study integrates SPE-based enrichment of the phenolic fraction with HPLC profiling and examines the antioxidant, antimicrobial, and microbial anti-adherence properties of the investigated honeys. However, the observed associations between specific phenolic acids and antioxidant potential, antimicrobial activity, or microbial anti-adherence effects should not be interpreted as evidence of causality, given the limitations of the present study outlined below.

The main limitations involve a restricted variety of honey types and an emphasis on specific phenolic acids. The phytochemical analysis omitted other bioactive compounds such as flavonoids, organic acids, enzymes, and methylglyoxal, which could also play a role—individually or synergistically—in the honey’s antioxidant and antimicrobial effects. Future research employing LC-MS/MS profiling and mechanistic studies may elucidate the precise functions of these components in the observed biological activities.

Each of the 12 honey types was represented by a single sample, which limits the generalizability of the results. Instead, the findings mainly showcase specific data and highlight differences. Additional research with larger sample sizes is needed to confirm these findings.

Additionally, biological effects were tested solely in vitro, and further in vivo research might be required to clarify the clinical significance of the observed bioactivities.

## 4. Materials and Methods

Phenolic acids in twelve honey varieties (black locust, multifloral, chestnut, thyme, pasture, manna, mint, hawthorn, rapeseed, linden, Manuka, and Tualang) were identified and quantified by high-performance liquid chromatography (HPLC). The antioxidant, antimicrobial, and anti-adherence activities of the honey samples were subsequently evaluated.

### 4.1. Reagents

High-purity reagents suitable for analytical applications were used throughout the study. HPLC-grade methanol (purity ≥ 99.9%) and acetonitrile of HPLC gradient grade (purity ≥ 99.9%) were purchased from Sigma-Aldrich, Merck KGaA (Darmstadt, Germany). Phosphoric acid suitable for HPLC (85%, LiChropur™) was obtained from the same supplier.

Ultrapure water (18.2 MΩ·cm), produced using a Milli-Q water purification system (Millipore, Bedford, MA, USA), was used throughout the study. Prior to analysis, all prepared samples were filtered through 0.45 µm Minisart^®^ Plus membrane filters (Teknokroma, Barcelona, Spain) to remove particulate matter.

Reference standards of 3-O-methylgallic acid (purity 99%) and ferulic acid (purity 99.2%) were obtained from ChromaDex and supplied by LGC Standards GmbH (Wesel, Germany). Gallic acid (purity ≥ 99%), caffeic acid (purity ≥ 98%), chlorogenic acid (purity ≥ 95%), *p*-coumaric acid (purity ≥ 98%), *trans*-resveratrol (purity ≥ 99%), syringic acid (purity ≥ 98.0%, HPLC), vanillin (purity 99%), ellagic acid (purity ≥ 95.0%, HPLC), and *trans*-cinnamic acid (purity ≥ 99%) were purchased from Sigma-Aldrich, Merck KGaA (Darmstadt, Germany). DPPH (purity ≥ 90%, elemental analysis), ABTS diammonium salt (purity ≥ 98%, HPLC), potassium persulfate (ACS reagent, purity ≥ 99.0%), and Trolox (purity 97%) were purchased from Sigma-Aldrich, Merck KGaA (Darmstadt, Germany).

### 4.2. Honey Samples

The samples were packed in glass containers and stored at room temperature (23 ± 1 °C) in the dark until analysis. In Table 12, the honey samples used and their vegetable sources are listed by geographical origin.

### 4.3. Solid-Phase Extraction

Phenolic acids were extracted from the honey samples using C18 solid-phase extraction (SPE) cartridges according to the method described by Chua et al. [55] with minor modifications. Briefly, each honey sample (5 g) was diluted with 25 mL of acidified distilled water. Solid-phase extraction was performed using LiChrolut^®^ RP-18 SPE cartridges (500 mg, 3 mL) (Merck KGaA, Darmstadt, Germany). The cartridges were attached to a vacuum manifold and sequentially conditioned with 3 mL each of methanol, acidified distilled water, and neutral distilled water. The diluted honey sample (30 mL) was loaded onto the preconditioned cartridge and passed at a dropwise flow rate. The cartridge was washed with 5 mL of acidified distilled water followed by 5 mL of neutral distilled water to remove residual sugars and interfering compounds. To remove residual moisture, the column was dried under vacuum for 5 min. Finally, the retained phenolic compounds were eluted dropwise with 5 mL of methanol.

It should be noted that highly hydrophilic phenolic acids, particularly gallic acid, may exhibit weak retention on the C18 sorbent and can be partially or completely lost during the aqueous washing steps, as previously observed by Chua et al. (2016) [55].

### 4.4. HPLC Analysis of Phenolic Acids

Separation was achieved on a Zorbax Eclipse XDB-C18 column (150 × 4.6 mm, 5 µm). The mobile phase consisted of solvent A (0.1% phosphoric acid in ultrapure water) and solvent B (acetonitrile) and was delivered using the following gradient program: 0–13 min, 90% A and 10% B; at 13 min, 78% A and 22% B; 14–17 min, 60% A and 40% B; at 17.5 min, 90% A and 10% B; and 17.5–22 min, 90% A and 10% B. The flow rate was 1.5 mL min^−1^, the column temperature was maintained at 35 °C, and the injection volume was 20 µL. Detection was performed at 310 nm using a diode-array detector [85].

The HPLC system consisted of an Agilent 1200 Series instrument equipped with a quaternary pump, diode array detector (DAD), online degasser, autosampler, and thermostated column compartment (Agilent Technologies, Santa Clara, CA, USA). Data acquisition and processing were performed using Agilent ChemStation software v.LTS 01.11 (Agilent Technologies, Santa Clara, CA, USA).

After SPE, a 5 mL aliquot of each solution was filtered through a 0.45 µm Millipore membrane filter (Millipore, Bedford, MA, USA) prior to HPLC-DAD analysis.

Standards (solutions in 70% methanol): *E*-resveratrol = 37 mg/mL, *Z*—resveratrol = 0.22 mg/mL, caffeic acid = 0.36 mg/mL, chlorogenic acid = 0.37 mg/mL, *trans*-cinnamic acid = 0.58 mg/mL, vanillin = 0.42 mg/mL, gallic acid = 0.39 mg/mL, 3-O-methylgallic acid = 0.33 mg/mL, ferulic acid = 0.50 mg/mL, ellagic acid = 0.28 mg/mL, syringic acid = 0.40 mg/mL. *Z*-Resveratrol was generated by photoisomerization of a *trans*-resveratrol solution under UV-C irradiation at 254 nm for 12 h, according to the procedure described by Stanciu et al. [86]. The Z isomer was identified based on its chromatographic retention time and DAD absorption spectrum, which were consistent with previously reported data for *cis*-resveratrol. Values are expressed as the mean ± SD (*n* = 6).

The mean retention times and corresponding standard deviations were calculated from six replicate injections (*n* = 6) using Microsoft Excel (Microsoft Corporation, Redmond, WA, USA). Phenolic compounds were identified by comparing their retention times and DAD absorption spectra with those of the corresponding reference standards. Chromatograms of the standard mixture and representative honey extracts are presented in Figures S3–S14 in the Supplementary Materials.

### 4.5. Analysis of Antioxidant Capacity

#### 4.5.1. DPPH Radical Scavenging Capacity Method

The antioxidant activity of the honey samples was evaluated using the DPPH radical scavenging assay. Honey stock extracts were prepared by dissolving 0.5 g of honey in 5 mL of 90% (*v*/*v*) methanol, yielding a final concentration of 100 mg/mL. Five working concentrations of 0.020, 0.035, 0.050, 0.065, and 0.080 g honey/mL were subsequently prepared [87].

For each concentration, 0.1 mL of honey extract was mixed with 3.9 mL of a freshly prepared 0.004% (*w*/*v*) DPPH methanolic solution. The mixtures were vortexed and incubated for 20 min at room temperature in the dark. Absorbance was measured at 517 nm using a UV–Vis spectrophotometer (V-630, JASCO, Tokyo, Japan). The control consisted of 0.1 mL of 90% methanol and 3.9 mL of DPPH solution, while methanol was used as the spectrophotometric blank.

The percentage of DPPH radical inhibition was calculated as follows [88]:

(1) $$DPPH\;inhibition\;(\%)\;=\;\frac{A_{control}\;-\;A_{sample}}{A_{control}}\;\times \;100$$

Trolox was used as the reference antioxidant. The calibration curve equation was y = 54.693x + 1.649, where y represents DPPH inhibition (%) and x represents the Trolox-equivalent concentration (µmol TE/mL). Antioxidant capacity was expressed as mg TE/g honey.

#### 4.5.2. ABTS Radical Scavenging Capacity Method

The antioxidant activity of the honey samples was also evaluated using the ABTS radical-scavenging assay. The method is based on the ability of antioxidant compounds present in honey to neutralize the blue–green ABTS^•+^ radical cation, resulting in a decrease in absorbance at 734 nm [87].

A 7 mM ABTS stock solution was prepared in deionized water and mixed with 2.45 mM potassium persulfate to generate the ABTS^•+^ radical cation. The mixture was incubated for 16 h at room temperature in the dark. Before use, the ABTS^•+^ solution was diluted with 10 mM phosphate buffer (pH 7.4) until an absorbance of 0.70 ± 0.02 at 734 nm was obtained.

Honey extracts were prepared in 90% (*v*/*v*) methanol at concentrations ranging from 0.2 to 1.0 mg/mL. For each concentration, 0.1 mL of honey extract was mixed with 2.9 mL of the ABTS^•+^ working solution, resulting in a final reaction volume of 3.0 mL. The mixtures were vortexed and incubated for 10 min at room temperature in the dark. Absorbance was measured at 734 nm using a UV–Vis spectrophotometer. The control consisted of 0.1 mL of 90% methanol and 2.9 mL of the ABTS^•+^ working solution, while methanol was used as the spectrophotometric blank.

The percentage of ABTS radical inhibition was calculated as follows [88]:

(2) $$ABTS\;inhibition\;(\%)\;=\;\frac{A_{control}\;-\;A_{sample}}{A_{control}}\;\times \;100$$

Trolox was used as the reference antioxidant. The equation of the Trolox calibration curve was y = 62.751x + 1.826, where y represents ABTS^•+^ radical inhibition (%) and x represents the Trolox-equivalent concentration expressed as µmol TE/mL. Antioxidant capacity was calculated from the calibration curve and expressed as mg TE/g honey.

### 4.6. Evaluation of Antimicrobial and Anti-Adherence Activities

The antimicrobial and anti-adherence activities of the honey extracts were evaluated using an agar diffusion assay for qualitative screening of growth inhibition, broth microdilution for quantitative determination of the minimum inhibitory concentration (MIC), and a semi-quantitative adherence assay to establish the minimum anti-adherence inhibitory concentration (MAIC). Microscopic examination was performed to assess microbial adherence qualitatively to an inert substrate.

The antimicrobial activity of the honey extracts was evaluated against the reference strains *E. faecalis* ATCC 29212, *S. aureus* ATCC 25923, *E. coli* ATCC 25922, *P. aeruginosa* ATCC 27853, and *C. albicans* ATCC 10231, as well as two clinical *C. albicans* isolates (24114 and 5329). All strains were obtained from the Microorganisms Collection of the Department of Microbiology, Faculty of Biology, and the Research Institute of the University of Bucharest.

#### 4.6.1. Qualitative Antimicrobial Assay

The qualitative antimicrobial activity of the honey extracts was assessed using an adapted agar diffusion assay, as previously described [CLSI] [89] with minor modifications [90]. Standardized microbial suspensions (1.5–3 × 10^8^ CFU/mL) were inoculated onto Mueller–Hinton agar (bacteria) or Sabouraud dextrose agar (*C. albicans*). 10 µL of each honey extract was applied to the inoculated agar surface, with methanol as the solvent control. Following a prediffusion period, the plates were incubated at 37 °C for 24 h; *C. albicans* plates were additionally evaluated after 48 h. Antimicrobial activity was expressed as the growth inhibition zone diameter (GIZD, mm).

#### 4.6.2. Minimum Inhibitory Concentration (MIC)

The MIC was determined by the broth microdilution method using sterile 96-well microplates, according to a previously reported protocol [89,90]. Serial two-fold dilutions of the honey extracts were prepared in the appropriate culture medium and inoculated with standardized microbial suspensions. Following incubation at 37 °C for 24 h, microbial growth was evaluated visually and spectrophotometrically at 620 nm using a Synergy HTX multimode microplate reader (BioTek Instruments, Winooski, VT, USA). The MIC was defined as the lowest concentration of honey extract required to inhibit visible microbial growth completely.

#### 4.6.3. Semiquantitative Assessment of Initial Microbial Adherence and Biofilm Formation

The inhibitory effect of the honey extracts on initial microbial adherence to an inert substrate was evaluated using the crystal violet microtiter plate assay, following a previously described protocol [91] with minor modifications. Briefly, serial twofold dilutions of the honey extracts were prepared in sterile 96-well microplates and inoculated simultaneously with standardized microbial suspensions, thereby allowing assessment of microbial adherence in the presence of the extracts from the start of the incubation period. After incubation at 37 °C for 24 h, the culture medium was removed, and the wells were washed 3 times with sterile distilled water to remove nonadherent cells. The adherent biomass was fixed with methanol for 10 min, stained with 1% (*w*/*v*) crystal violet for 15 min, and subsequently solubilized with 33% (*v*/*v*) acetic acid. The absorbance was measured at 490 nm using a Synergy HTX multimode microplate reader (BioTek Instruments, Winooski, VT, USA). The minimum adherence inhibitory concentration (MAIC) was defined as the lowest concentration of honey extract required to prevent microbial adherence to the inert surface, as determined by the crystal violet assay.

### 4.7. Statistical Analysis

Statistical analyses were performed using SigmaXL: Version 11.03 (2026) (SigmaXL Inc., Kitchener, ON, Canada). Experimental data are presented as mean ± standard deviation (SD) across three independent determinations unless otherwise stated.

Statistically significant differences between variables were assessed using Welch’s ANOVA with a Games–Howell post hoc test.

Pearson correlation analyses were used to evaluate the correlations among phenolic acids, antioxidant activity, and antimicrobial activity. Correlation coefficients (*r*) and corresponding *p*-values were calculated, and statistical significance was established at *p* < 0.05, with 95% confidence interval (CI). The linear correlation level was interpreted as follows: strong correlation (0.8 ≤ r < 1); moderate correlation (0.4 ≤ r < 0.8); and low correlation (0 < r < 0.4) [92]. Scatter plots with linear regression models were generated to visualize the relationships between selected variables identified as statistically significant by Pearson correlation analysis.

Multiple Regression was conducted to examine the relationship between a single dependent variable (Y) and multiple independent variables (Xs) and to clarify how these variables interact. SigmaXL estimates the model coefficients and the constant term using the least squares method (R-Square, R-Square Adjusted, and Root mean square error). It also provides hypothesis tests for the model coefficients. To assess multicollinearity among predictors, the variance inflation factor (VIF) and Tolerance (T) scores are used. A VIF of 1 indicates no correlation among predictors (which is ideal and common in Design of Experiments). VIF values > 1 suggest some multicollinearity, whereas VIF values > 5 indicate strong multicollinearity, potentially leading to unreliable coefficient estimates. Tolerance is calculated as 1/VIF. The Durbin–Watson Test is used to determine if the residuals are autocorrelated. If either *p*-value is <0.05, then there is significant autocorrelation. These approaches were detailed in the Supplementary Materials.

## 5. Conclusions

This study demonstrates that 12 honey varieties from various botanical and geographic origins exhibit distinct phenolic acid profiles, antioxidant potential, antimicrobial efficacy, and the ability to inhibit microbial adherence. A notable aspect of this research is the combination of SPE-based phenolic enrichment with HPLC profiling and pharmacological assessment. The findings indicate that honey’s source impacts its phytochemical composition and biological functions, revealing potential bioactive compounds that may explain its pharmacological properties. The main limitations include the relatively small sample size and the focus on select phenolic acids. Future studies should involve larger, more diverse samples, expand chemical analyses, isolate and test specific compounds and their combinations, and utilize in vivo models to better understand the individual and synergistic effects and their biological significance.

## Figures and Tables

**Table 1:** Retention times and analytical performance parameters of the HPLC-DAD method for the investigated phenolic compounds.

No.	Phenolic Compound	Retention Time ± SD (min)	Liniar Range (μg/mL)	Calibration Equation	R^2^	LOD (µg/mL)	LOQ (µg/mL)
1	gallic acid	0.958 ± 0.025	2–100	y = 2.9889x + 1.5423	0.9996	0.5150	7.4177
2	3-O-methylgallic acid	2.606 ± 0.008	2–100	y = 5.7013x − 1.3292	0.9972	1.3330	4.0400
3	chlorogenic acid	3.501 ± 0.015	2–100	y = 25.1743x + 12.249	0.9999	0.3730	1.9109
4	caffeic acid	4.597 ± 0.036	2–100	y = 38.688x − 4.727	0.9961	0.3300	1.0100
5	syringic acid	5.054 ± 0.021	2–100	y = 25.541x − 4.3533	0.9984	0.9762	1.0074
6	vanillin	6.898 ± 0.051	2–100	y = 36.869x − 2.1499	0.9969	0.4355	0.9552
7	*p*-coumaric acid	7.187 ± 0.019	2–100	y = 40.596x − 0.3492	0.9953	1.1450	3.4714
8	ferulic acid	8.567 ± 0.058	2–100	y = 6.8472x − 6.3527	0.9986	1.1020	3.3360
9	*E*-resveratrol	14.409 ± 0.017	2–100	y = 30.893x + 19.054	0.9988	1.1070	3.3545
10	ellagic acid	15.303 ± 0.027	2–100	y = 10.069x + 51.897	0.9979	3.2705	9.9106
11	*Z*-resveratrol	15.751 ± 0.058	2–100	y = 32.428x − 2.5436	0.9956	0.0509	0.9378
12	*trans*-cinnamic acid	15.867 ± 0.007	2–100	y = 9.027x + 0.5635	0.9999	0.1762	0.5539

**Table 2:** The HPLC-DAD results of phenolic constituents concentration (µg/g honey) in the alcoholic extract of honey samples (expressed as a mean ± standard deviation; *n* = 3).

Sample	Chlorogenic Acid	Caffeic Acid	Ferulic Acid	*trans*-Cinnamic Acid	3-O-Methylgallic Acid	*p*-Coumaric Acid	Syringic Acid	Vanillin
P1	n.d.	0.81 ± 0.02	0.86 ± 0.04	6.10 ± 0.14	n.d.	1.57 ± 0.07	n.d.	n.d.
P2	n.d.	22.99 ± 0.45	n.d.	18.02 ± 0.39	152.87 ± 3.42	0.69 ± 0.04	26.74 ± 0.41	n.d.
P3	39.79 ± 0.18	n.d.	n.d.	20.53 ± 0.52	n.d.	n.d.	1.32 ± 0.07	0.15 ± 0.02
P4	1.31 ± 0.04	1.38 ± 0.04	2.47 ± 0.08	13.90 ± 0.36	5.53 ± 0.22	4.22 ± 0.14	n.d.	n.d.
P5	0.57 ± 0.02	0.57 ± 0.02	n.d.	4.61 ± 0.15	6.15 ± 0.25	0.70 ± 0.04	n.d.	n.d.
P6	1.76 ± 0.04	0.90 ± 0.03	0.65 ± 0.04	10.32 ± 0.33	2.96 ± 0.13	1.17 ± 0.06	0.09 ± 0.01	n.d.
P7	1.92 ± 0.04	0.98 ± 0.03	3.66 ± 0.12	13.31 ± 0.35	2.66 ± 0.14	5.71 ± 0.18	0.09 ± 0.02	n.d.
P8	4.09 ± 0.07	0.44 ± 0.03	2.98 ± 0.09	8.21 ± 0.23	10.37 ± 0.37	6.48 ± 0.22	n.d.	n.d.
P9	128.69 ± 0.45	33.25 ± 0.45	1.67 ± 0.06	8.06 ± 0.28	206.29 ± 4.56	n.d.	n.d.	n.d.
P10	2.24 ± 0.05	1.78 ± 0.06	n.d.	13.17 ± 0.33	n.d.	1.60 ± 0.08	n.d.	n.d.
P11	4.81 ± 0.07	3.98 ± 0.10	0.67 ± 0.04	19.78 ± 0.56	n.d.	1.42 ± 0.07	0.16 ± 0.02	n.d.
P12	1.16 ± 0.03	4.05 ± 0.10	n.d.	n.d.	n.d.	n.d.	n.d.	n.d.

n.d. = Not detected under the conditions of the method used. P1: black locust honey; P2: multiflower honey; P3: chestnut honey; P4: thyme honey; P5: pasture honey; P6: manna honey; P7: mint honey; P8: hawthorn honey; P9: rapeseed honey; P10: linden honey; P11: Manuka honey; P12: Tualang honey.

**Table 3:** The statistically significant differences between phenolic acid contents detected in tested honey varieties.

**chlorogenic acid**																			
***p*-values**	**P11**		**P12**				**P3**	**P4**		**P5**		**P6**		**P7**		**P8**		**P9**	
**P10**	0.0000		0.0003				0.0000	0.0003		0.0003		0.0027		0.0119		0.0001		0.0000	
**P11**			0.0001				0.0000	0.0000		0.0001		0.0000		0.0000		0.0024		0.0000	
**P12**							0.0000	0.0516		0.0003		0.0005		0.0002		0.0001		0.0000	
**P3**								0.0000		0.0000		0.0000		0.0000		0.0000		0.0000	
**P4**										0.0005		0.0016		0.0005		0.0001		0.0000	
**P5**												0.0002		0.0001		0.0002		0.0000	
**P6**														0.0736		0.0001		0.0000	
**P7**																0.0001		0.0000	
**P8**																		0.0000	
**caffeic acid**																			
***p*-values**	**P10**	**P11**			**P12**	**P2**		**P4**		**P5**		**P6**		**P7**		**P8**		**P9**	
**P1**	0.0030	0.0011			0.0011	0.0007		0.0020		0.0014		0.1423		0.0208		0.0015		0.0003	
**P10**		0.0003			0.0003	0.0006		0.0121		0.0018		0.0017		0.0023		0.0005		0.0002	
**P11**					0.9931	0.0005		0.0006		0.0010		0.0007		0.0008		0.0005		0.0002	
**P12**						0.0005		0.0006		0.0009		0.0007		0.0007		0.0005		0.0002	
**P2**								0.0007		0.0007		0.0007		0.0007		0.0006		0.0001	
**P4**										0.0007		0.0014		0.0027		0.0001		0.0003	
**P5**												0.0023		0.0011		0.0474		0.0003	
**P6**														0.2668		0.0006		0.0003	
**P7**																0.0003		0.0003	
**P8**																		0.0003	
***trans*-cinnamic acid**																			
***p*-values**	**P10**	**P11**			**P2**	**P3**		**P4**		**P5**		**P6**		**P7**		**P8**			**P9**
**P1**	0.0009	0.0017			0.0005	0.0011		0.0011		0.0027		0.0036		0.0012		0.0048			0.0146
**P10**		0.0023			0.0011	0.0011		0.4413		0.0004		0.0052		0.9999		0.0007			0.0005
**P11**					0.1253	0.7910		0.0028		0.0012		0.0007		0.0022		0.0011			0.0006
**P2**						0.0339		0.0021		0.0003		0.0002		0.0012		0.0002			0.0001
**P3**								0.0013		0.0008		0.0004		0.0010		0.0006			0.0003
**P4**										0.0006		0.0027		0.6510		0.0007			0.0004
**P5**												0.0014		0.0006		0.0007			0.0025
**P6**														0.0049		0.0135			0.0102
**P7**																0.0009			0.0006
**P8**																			0.9980
**ferulic acid**																			
***p*-values**	**P4**			**P5**			**P6**			**P7**		**P8**				**P9**			
**P2**	0.0006			0.0006			0.0006			0.0006		0.0006				0.0009			
**P4**				0.1894			0.0015			0.0009		0.0011				0.0006			
**P5**							0.0016			0.0010		0.0012				0.0006			
**P6**										0.2861		0.0012				0.0006			
**P7**												0.0009				0.0006			
**P8**																0.0006			
***p*-coumaric acid**																			
***p*-values**	**P10**				**P11**		**P2**		**P4**		**P5**		**P6**			**P7**	**P8**		
**P1**	0.9996				0.3782		0.0017		0.0007		0.0018		0.0164			0.0008	0.0013		
**P10**					0.2975		0.0031		0.0005		0.0032		0.0196			0.0006	0.0010		
**P11**							0.0031		0.0006		0.0033		0.0816			0.0007	0.0012		
**P2**									0.0012		1.0000		0.0055			0.0012	0.0016		
**P4**											0.0012		0.0007			0.0042	0.0025		
**P5**													0.0059			0.0012	0.0016		
**P6**																0.0009	0.0013		
**P7**																	0.0825		
**3-O-methylgallic acid**																			
***p*-values**	**P4**				**P5**		**P6**			**P7**		**P8**				**P9**			
**P2**	0.0006				0.0006		0.0006			0.0006		0.0006				0.0009			
**P4**					0.1894		0.0015			0.0009		0.0011				0.0006			
**P5**							0.0016			0.0010		0.0012				0.0006			
**P6**										0.2861		0.0012				0.0006			
**P7**												0.0009				0.0006			
**P8**																0.0006			
**syringic acid**																			
***p*-values**	**P2**					**P3**				**P6**					**P7**				
**P11**	0.0002					0.0019				0.0484					0.0576				
**P2**						0.0001				0.0002					0.0002				
**P3**										0.0027					0.0016				
**P6**															1.0000				

Red *p*-values are statistically significant (*p* < 0.05). CI = 95%. P1: black locust honey; P2: multiflower honey; P3: chestnut honey; P4: thyme honey; P5: pasture honey; P6: manna honey; P7: mint honey; P8: hawthorn honey; P9: rapeseed honey; P10: linden honey; P11: Manuka honey; P12: Tualang honey.

**Table 4:** The antioxidant activity of the honey extracts determined by the DPPH assay.

Sample	Antioxidant Activity (AA) % *	Antioxidant Capacity (mg TE/g Honey)
P1	22.091 ± 2.144	0.929
P2	29.966 ± 0.930	1.294
P3	40.077 ± 2.045	1.677
P4	27.206 ± 3.843	1.153
P5	28.506 ± 1.815	1.188
P6	28.100 ± 2.447	1.193
P7	19.766 ± 2.220	0.827
P8	21.055 ± 3.276	0.884
P9	39.524 ± 3.7413	1.703
P10	11.466 ± 5.968	0.448
P11	29.912 ± 2.242	1.292
P12	49.248 ± 1.963	2.103

* Expressed as mean ± SD (*n* = 3); TE, Trolox equivalents. P1: black locust honey; P2: multiflower honey; P3: chestnut honey; P4: thyme honey; P5: pasture honey; P6: manna honey; P7: mint honey; P8: hawthorn honey; P9: rapeseed honey; P10: linden honey; P11: Manuka honey; P12: Tualang honey.

**Table 5:** The antioxidant activity of the honey extracts determined by the ABTS assay.

Sample	Antioxidant Activity (AA)% *	Antioxidant Capacity (mg TE/g Honey)
P1	21.779 ± 2.512	0.768
P2	34.884 ± 4.797	1.275
P3	34.939 ± 2.422	1.315
P4	24.863 ± 4.589	0.903
P5	31.515 ± 2.296	1.153
P6	27.774 ± 2.170	1.032
P7	19.124 ± 2.863	0.685
P8	21.090 ± 2.753	0.757
P9	41.900 ± 5.086	1.578
P10	18.721 ± 2.210	0.671
P11	33.972 ± 3.226	1.223
P12	44.560 ± 1.116	1.701

* Expressed as mean ± SD; SD (*n* = 3). TE, Trolox equivalents. P1: black locust honey; P2: multiflower honey; P3: chestnut honey; P4: thyme honey; P5: pasture honey; P6: manna honey; P7: mint honey; P8: hawthorn honey; P9: rapeseed honey; P10: linden honey; P11: Manuka honey; P12: Tualang honey.

**Table 6:** The significant differences between antioxidant activities (%) of different honey varieties determined by DPPH and ABTS assays.

*p*-Values	P10	P11	P12	P2	P3	P4	P5	P6	P7	P8	P9
**DPPH radical scavenging activity**											
**P1**	0.1618	0.0117	0.0000	0.0076	0.0000	0.4197	0.0223	0.0679	0.8361	0.9807	0.0015
**P10**		0.0188	0.0008	0.0249	0.0028	0.0340	0.0289	0.0283	0.3342	0.3913	0.0013
**P11**			0.0000	1.0000	0.0019	0.9385	0.9858	0.9706	0.0024	0.0355	0.0380
**P12**				0.0000	0.0023	0.0006	0.0000	0.0000	0.0000	0.0001	0.0359
**P2**					0.0016	0.8671	0.8615	0.8599	0.0026	0.0432	0.0470
**P3**						0.0103	0.0004	0.0010	0.0000	0.0009	1.0000
**P4**							0.9995	1.0000	0.1300	0.2549	0.0204
**P5**								1.0000	0.0040	0.0698	0.0204
**P6**									0.0119	0.0915	0.0158
**P7**										1.0000	0.0007
**P8**											0.0010
**ABTS reducing power**											
**P1**	0.6653	0.0049	0.0001	0.0285	0.0008	0.9473	0.0054	0.0781	0.8847	1.0000	0.0046
**P10**		0.0012	0.0000	0.0110	0.0001	0.3909	0.0005	0.0047	1.0000	0.9035	0.0026
**P11**			0.0152	1.0000	1.0000	0.1334	0.9345	0.1436	0.0016	0.0039	0.2923
**P12**				0.1090	0.0049	0.0060	0.0007	0.0001	0.0001	0.0001	0.9716
**P2**					1.0000	0.1655	0.9205	0.2895	0.0107	0.0218	0.5641
**P3**						0.0759	0.5387	0.0259	0.0004	0.0009	0.3686
**P4**							0.3217	0.9559	0.5102	0.8730	0.0130
**P5**								0.3705	0.0021	0.0053	0.1005
**P6**									0.0186	0.0623	0.0280
**P7**										0.9846	0.0020
**P8**											0.0035

Red *p*-values are statistically significant (*p* < 0.05). CI = 95%. P1: black locust honey; P2: multiflower honey; P3: chestnut honey; P4: thyme honey; P5: pasture honey; P6: manna honey; P7: mint honey; P8: hawthorn honey; P9: rapeseed honey; P10: linden honey; P11: Manuka honey; P12: Tualang honey.

**Table 7:** The differences between antimicrobial activities (GIZD, mm) of different honey varieties.

***P. aeruginosa* ATCC 27853**																												
***p*-values**	**P10**		**P11**			**P12**			**P2**				**P3**				**P4**		**P5**		**P6**		**P7**		**P8**			**P9**
**P1**	1.0000		1.0000			0.9973			0.9964				0.9994				1.0000		0.9998		1.0000		1.0000		1.0000			1.0000
**P10**			1.0000			0.9986			0.9977				0.9998				1.0000		1.0000		1.0000		0.9998		1.0000			1.0000
**P11**						0.9767			0.9749				0.9930				1.0000		0.9978		0.9999		1.0000		1.0000			1.0000
**P12**									1.0000				1.0000				0.9947		1.0000		1.0000		0.9622		0.9973			0.9944
**P2**													1.0000				0.9934		1.0000		1.0000		0.9613		0.9965			0.9926
**P3**																	0.9986		1.0000		1.0000		0.9851		0.9993			0.9990
**P4**																			0.9995		1.0000		1.0000		1.0000			1.0000
**P5**																					1.0000		0.9945		0.9998			0.9997
**P6**																							0.9992		1.0000			1.0000
**P7**																									1.0000			1.0000
**P8**																												1.0000
***E. faecalis* ATCC 29212**																												
***p*-values**	**P10**				**P11**		**P12**			**P2**					**P4**			**P5**		**P6**			**P7**	**P8**				**P9**
**P1**	0.4464				0.4162		0.0402			0.6109					0.5874			0.1449		0.4834			0.3625	0.3499				0.3499
**P10**					0.9412		0.7185			0.9654					1.0000			0.5080		0.9588			0.8739	0.9896				0.9896
**P11**							1.0000			1.0000					0.8739			0.9987		1.0000			1.0000	1.0000				1.0000
**P12**										1.0000					0.5560			0.9459		1.0000			1.0000	0.9998				0.9998
**P2**															0.9295			1.0000		1.0000			1.0000	0.9998				0.9998
**P4**																		0.4095		0.9065			0.7880	0.9474				0.9474
**P5**																				0.9992			1.0000	0.9175				0.9175
**P6**																							1.0000	1.0000				1.0000
**P7**																								0.9987				0.9987
**P8**																												1.0000
***C. albicans* 10231**																												
***p*-values**	**P10**		**P11**			**P12**			**P2**					**P3**			**P4**		**P5**			**P6**	**P7**			**P8**		**P9**
**P1**	0.7367		0.3552			0.3552			0.5142					0.6410			0.9835		0.9993			1.0000	0.7742			1.0000		0.9999
**P10**			0.9835			0.1889			0.2293					0.1482			0.5795		0.8026			0.6942	0.3998			0.8376		0.9967
**P11**						0.1673			0.1873					0.0861			0.4079		0.6263			0.1367	0.3108			0.6420		0.9002
**P12**									0.9981					0.7742			0.7367		0.7493			0.3108	0.9805			0.6410		0.3383
**P2**														0.9835			0.9556		0.9477			0.4079	1.0000			0.8796		0.5130
**P3**																	0.9999		0.9993			0.3552	1.0000			0.9911		0.6942
**P4**																			1.0000			0.9002	0.9981			1.0000		0.9556
**P5**																						0.9879	0.9957			1.0000		0.9931
**P6**																							0.6420			0.9969		1.0000
**P7**																										0.9805		0.7367
**P8**																												0.9981
***C. albicans* 24114**																												
***p*-values**		**P10**		**P11**				**P12**			**P2**					**P4**			**P5**		**P7**		**P8**				**P9**	
**P1**		0.2731		0.1867				0.8630			0.3450					1.0000			0.1897		0.3591		0.0108				0.3445	
**P10**				1.0000				0.8041			0.9999					0.6586			0.9996		0.9985		0.9953				0.6107	
**P11**								0.8397			0.9954					0.6955			0.9852		0.9833		0.8898				0.5619	
**P12**											0.7272					0.9930			0.5796		0.6916		0.4060				1.0000	
**P2**																0.5881			1.0000		1.0000		1.0000				0.6116	
**P4**																			0.5123		0.5562		0.4612				0.9791	
**P5**																					1.0000		1.0000				0.4060	
**P7**																							1.0000				0.5990	
**P8**																											0.0464	
***C. albicans* 5329**																												
***p*-values**		**P10**		**P12**				**P2**				**P3**				**P4**			**P6**		**P7**		**P8**				**P9**	
**P1**		0.6444		0.4616				0.1280				0.9928				0.9758			0.4499		0.2579		0.4616				0.3995	
**P10**				0.9714				0.3191				1.0000				0.9996			0.8630		0.4581		0.9714				0.8898	
**P12**								0.6841				0.9928				1.0000			0.9984		0.6484		1.0000				1.0000	
**P2**												0.6444				1.0000			0.9852		0.9954		0.6841				0.8749	
**P3**																0.9992			0.9482		0.5549		0.9928				0.9714	
**P4**																			1.0000		0.9975		1.0000				1.0000	
**P6**																					0.8962		0.9984				1.0000	
**P7**																							0.6484				0.7638	
**P8**																											1.0000	

Red *p*-values are statistically significant (*p* < 0.05). CI = 95%. P1: black locust honey; P2: multiflower honey; P3: chestnut honey; P4: thyme honey; P5: pasture honey; P6: manna honey; P7: mint honey; P8: hawthorn honey; P9: rapeseed honey; P10: linden honey; P11: Manuka honey; P12: Tualang honey.

**Table 8:** Pearson correlation between antioxidant activity and phenolic constituents quantified in honey varieties.

Pearson Correlation	ABTS AA%	Chl.A.	C.A.	F.A.	t-Cin.A	3-O-MGA	p-Cou.A	SA	Van
**Correlation Coefficient (*r*)**									
DPPH AA%	0.9327	0.4157	0.3422	−0.3335	−0.1941	0.2837	−0.5849	0.0488	0.3435
ABTS AA%	1	0.4869	0.5475	−0.4752	−0.1397	0.4781	−0.7188	0.2004	0.1925
chlorogenic acid		1	0.7330	0.0755	−0.0136	0.7190	−0.3363	−0.1229	0.2048
caffeic acid			1	−0.0589	0.0418	0.9883	−0.3727	0.4958	−0.1749
ferulic acid				1	−0.0078	−0.0170	0.8748	−0.2732	−0.2603
*trans*-cinnamic acid					1	0.0598	0.0468	0.3610	0.4594
3-O-methylgallic acid						1	−0.3132	0.5375	−0.1454
*p*-coumaric acid							1	−0.1920	−0.2752
syringic acid								1	−0.0429
***p*-values**									
DPPH AA%	0.0000	0.1790	0.2762	0.2894	0.5456	0.3715	0.0457	0.8802	0.2743
ABTS AA%		0.1084	0.0654	0.1185	0.6649	0.1160	0.0084	0.5323	0.5489
chlorogenic acid			0.0067	0.8156	0.9666	0.0084	0.2852	0.7037	0.5231
caffeic acid				0.8557	0.8973	0.0000	0.2328	0.1012	0.5866
ferulic acid					0.9807	0.9582	0.0002	0.3902	0.4139
*trans*-cinnamic acid						0.8536	0.8850	0.2489	0.1329
3-O-methylgallic acid							0.3216	0.0715	0.6522
*p*-coumaric acid								0.5500	0.3866
syringic acid									0.8947

*r* = correlation coefficient (Pearson); Colored *r* values are statistically significant, *p* < 0.05; *p*-values in red are < 0.05. The correlation level was interpreted as follows: strong (0.8 ≤ *r* < 1), moderate (0.4 ≤ *r* < 0.8), and low (0 < *r* < 0.4). AA—antioxidant activity (%), ChlA—chlorogenic acid, CA—caffeic acid, FA—ferulic acid, *t*-CinA—*trans*-cinnamic acid, 3-O-MGA—3-O-methylgallic acid, *p*-CouA—*p*-coumaric acid, SA—syringic acid.

**Table 9:** Pearson correlation between antimicrobial activity (expressed as MIC) and phenolic constituents quantified in honey varieties.

Pearson Correlation	MIC C.a. ATCC 10231	MIC C.a.24114	MIC C.a.5329	MIC P.a. ATCC 27853	MIC E.f. ATCC 29212
**Correlation Coefficient (*r*)**					
chlorogenic acid	0.3581	0.5002	0.1207	0.7086	0.5701
caffeic acid	0.2110	0.4656	0.0566	0.7206	0.3595
ferulic acid	0.2233	0.5303	0.2613	0.1720	0.0900
*trans*-cinnamic acid	−0.1090	−0.1627	0.5693	−0.1255	−0.2118
3-O-methylgallic acid	0.2484	0.5104	0.1473	0.7223	0.3680
*p*-coumaric acid	0.2101	0.3927	0.2792	−0.1131	−0.1914
syringic acid	−0.1788	−0.0743	0.1037	0.0182	−0.2810
vanillin	−0.1676	−0.3898	0.0973	−0.3632	−0.1717
***p*-values**					
chlorogenic acid	0.2531	0.0977	0.7086	0.0099	0.0530
caffeic acid	0.5104	0.1271	0.8613	0.0082	0.2510
ferulic acid	0.4854	0.0761	0.4119	0.5930	0.7809
*trans*-cinnamic acid	0.7359	0.6135	0.0534	0.6975	0.5087
3-O-methylgallicacid	0.4363	0.0900	0.6477	0.0080	0.2393
*p*-coumaric acid	0.5122	0.2067	0.3795	0.7264	0.5513
syringic acid	0.5782	0.8185	0.7484	0.9552	0.3764
vanillin	0.6027	0.2103	0.7634	0.2459	0.5935

*r* = correlation coefficient (Pearson); colored *r* values are statistically significant, *p* < 0.05; *p*-values in red are < 0.05. The linear correlation level was interpreted as follows: strong (0.8 ≤ *r* < 1), moderate (0.4 ≤ *r* < 0.8), and low (0 < *r* < 0.4). C.a. = *Candida albicans*, P.a. = *Pseudomonas aeruginosa*, E.f. = *Enterococcus faecalis*, MIC = Minimum Inhibitory Concentration (g/mL).

**Table 10:** Pearson correlation between phenolic constituents and the inhibitory activity of microbial adherence to an inert substrate of all honey samples P1–12.

Pearson Correlation	MAIC C.a. ATCC 10231	MAIC C.a.24114	MAIC C.a.5329	MAIC P.a. ATCC 27853	MAIC E.f. ATCC 29212
**Correlation Coefficient (*r*)**					
chlorogenic acid	0.3565	0.2628	0.2822	0.4464	0.7888
caffeic acid	0.1984	0.3144	0.1148	0.3242	0.6057
ferulic acid	0.6915	0.2661	0.6081	−0.0239	0.2162
*trans*-cinnamic acid	−0.1057	0.0086	−0.1262	−0.0282	−0.2947
3-O-methylgallic acid	0.2547	0.2628	0.1769	0.3280	0.6336
*p*-coumaric acid	0.5159	0.2488	0.4366	−0.1399	−0.0862
syringic acid	−0.1768	−0.1392	−0.2161	−0.1386	−0.1628
vanillin	−0.1676	−0.4989	−0.2060	−0.1298	−0.1526
***p*-values**					
chlorogenic acid	0.2553	0.4092	0.3742	0.1457	0.0023
caffeic acid	0.5366	0.3195	0.7223	0.3040	0.0368
ferulic acid	0.0127	0.4032	0.0359	0.9413	0.4997
*trans*-cinnamic acid	0.7438	0.9788	0.6959	0.9308	0.3525
3-O-methylgallic acid	0.4244	0.4092	0.5824	0.2979	0.0269
*p*-coumaric acid	0.0860	0.4356	0.1559	0.6644	0.7899
syringic acid	0.5824	0.6662	0.5000	0.6676	0.6132
vanillin	0.6027	0.0987	0.5207	0.6875	0.6359

*r* = correlation coefficient (Pearson); colored *r* values are statistically significant, *p* < 0.05; *p*-values in red are < 0.05. The linear correlation level was interpreted as follows: strong (0.8 ≤ *r* < 1), moderate (0.4 ≤ *r* < 0.8), and low (0 < *r* < 0.4). C.a. = *Candida albicans*, P.a. = *Pseudomonas aeruginosa*, E.f. = *Enterococcus faecalis*, MAIC = Minimum Adherence Inhibitory Concentration (g/mL); red values are statistically significant (*p* < 0.05).

**Table 11:** Pearson Correlation between reducing sugars, HMF, diastase activity, and antimicrobial activity of all honey samples P1-12.

Pearson Correlation	DPPH AA%	ABTS AA%	MIC C.a. ATCC 10231	MIC C.a.24114	MIC C.a.5329	MIC P.a. ATCC 27853	MIC E.f. ATCC 29212
**Correlation Coefficient (*r*)**							
Red sugars	−0.0765	0.1117	0.0206	0.4370	0.0490	0.3985	−0.0207
Diastase index	−0.0756	−0.1340	0.0517	0.0405	0.3661	0.0656	0.5047
HMF	0.5462	0.5230	−0.5890	−0.4207	−0.8258	−0.3152	−0.1716
DPPH AA%	1	0.9327	−0.5185	−0.2470	−0.6248	−0.0971	0.1415
ABTS AA%		1	−0.4291	−0.1422	−0.5388	0.1175	0.2124
***p*-values**							
Red sugars	0.8133	0.7296	0.9493	0.1555	0.8797	0.1994	0.9492
Diastase index	0.8153	0.6781	0.8731	0.9005	0.2418	0.8395	0.0942
HMF	0.0662	0.0810	0.0439	0.1732	0.0009	0.3183	0.5938
DPPH AA%		0.0000	0.0842	0.4390	0.0298	0.7641	0.6609
ABTS AA%			0.1639	0.6593	0.0707	0.7162	0.5074

HMF –5—hydroxymethylfurfural; *r* = correlation coefficient (Pearson); colored *r* values are statistically significant; *p*-values in red are < 0.05. The linear correlation level was interpreted as follows: strong (0.8 ≤ *r* < 1), moderate (0.4 ≤ *r* < 0.8), and low (0 < *r* < 0.4). C.a. = *Candida albicans*, P.a. = *Pseudomonas aeruginosa*, E.f. = *Enterococcus faecalis*, MIC = minimum inhibitory concentration.

**Table 12:** Samples of honey used and plant source according to geographical origin.

Honey Type	Provenance	Source
Black locust honey (P1)	Romania	*Robinia* sp.
Multiflower honey (P2)	Romania	mixed floral sources *
Chestnut honey (P3)	Spain	*Castanea* sp.
Thyme honey (P4)	Romania	*Thymus* sp.
Pasture honey (P5)	Romania	mixed floral sources **
Manna honey (P6)	Romania	forest honeydew origin
Mint honey (P7)	Romania	*Mentha* sp.
Hawthorn honey (P8)	Romania	*Crataegus* sp.
Rapeseed honey (P9)	Romania	*Brassica* sp.
Linden honey (P10)	Romania	*Tilia* sp.
Manuka honey (P11)	New Zealand	*Leptospermum* sp.
Tualang honey (P12)	Malaysia	*Koompassia* sp.

* Brassicaceae 42.74%, Fabaceae 27.43%, Asteraceae 9.15% [53]. ** Rosaceae 69.38%, Fabaceae 18.57% [53].

## Data Availability

The original contributions presented in the study are included in the article, further inquiries can be directed to the corresponding authors.

## Supplementary Materials

The following supporting information can be downloaded at: https://www.mdpi.com/article/10.3390/ijms27177848/s1.

## Abbreviations

The following abbreviations are used in this manuscript:

ABTS	2,2-azino-bis-3-ethylbenzothiazoline-6-sulphonic acid
ATCC	American Type Culture Collection
CFU	Colony-Forming Units
DPPH	2,2′-diphenyl-1-picrylhydrazyl
TE	Trolox Equivalents
GIZD	Growth Inhibition Zone Diameter
HMF	5-Hydroxymethylfurfural
HPLC-DAD	High-Performance Liquid Chromatography with Diode Array Detection
MAIC	Minimum Adherence Inhibitory Concentration
MIC	Minimum Inhibitory Concentration
PAP	Phenolic Acid Profile
R^2^	Coefficient of Determination
SD	Standard Deviation
SPE	Solid-Phase Extraction
CI	Confidence interval
*r*	Correlation coefficient (Pearson)
VIF	Variance inflation factor
*T*	Tolerance score
DW	Durbin–Watson autocorrelation
Mult Reg	Multiple Regression
Residuals	Unexplained variations from the regression model
