# Supplementary material for: Comparative Analysis of Phenolic Acid Profiles, Antioxidant Capacity, and Antimicrobial Activity of Honeys from Different Botanical and Geographical Origins
Source: Int J Mol Sci. 2026 Sep 2;27(17):7848. doi: 10.3390/ijms27177848 (PMC13565909; doi:10.3390/ijms27177848)
Supplement: Supplementary file 1 [file ijms-27-07848-s001.zip › ijms-4513033-supplementary material Figures 1 - 14.pdf]

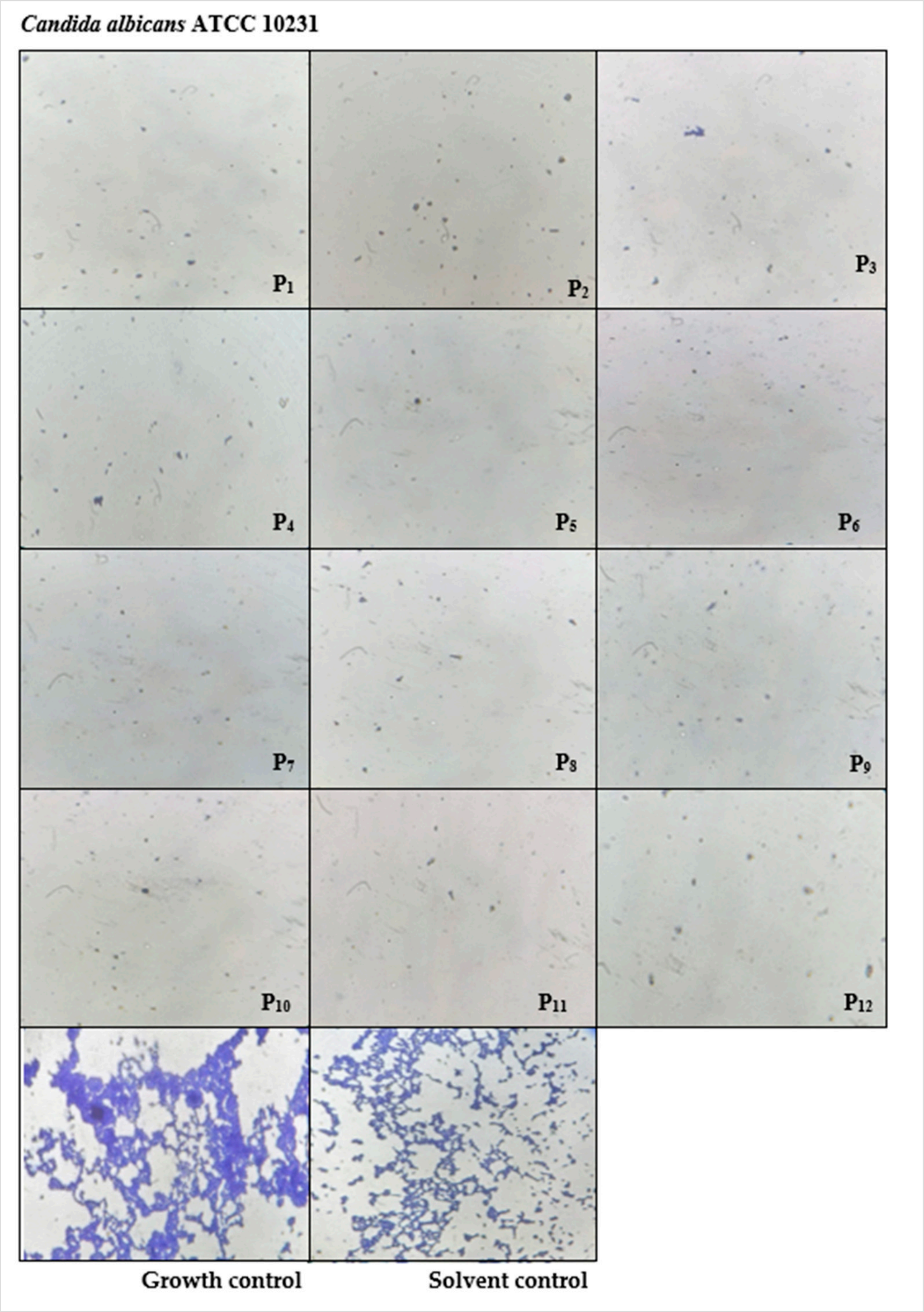

Figure S1. Optical microscopy images of fungal adherence in *C. albicans* strain ATCC 10231 in the presence of the honey extracts (inverted microscope, 400x magnification).

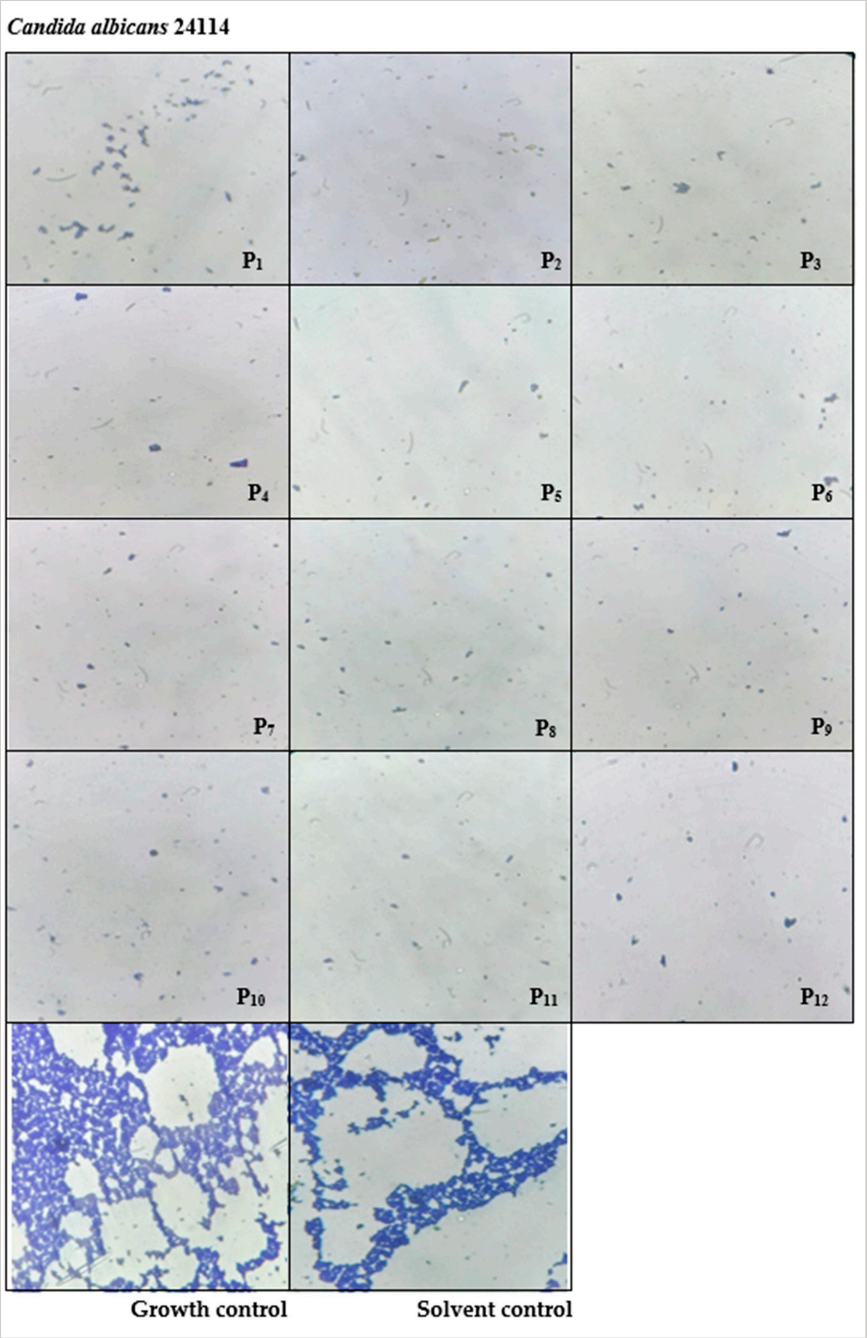

Figure S2. Optical microscopy images of the fungal adherence in *C. albicans* strain 24114 in the presence of the honey extracts (inverted microscope, 400x magnification).

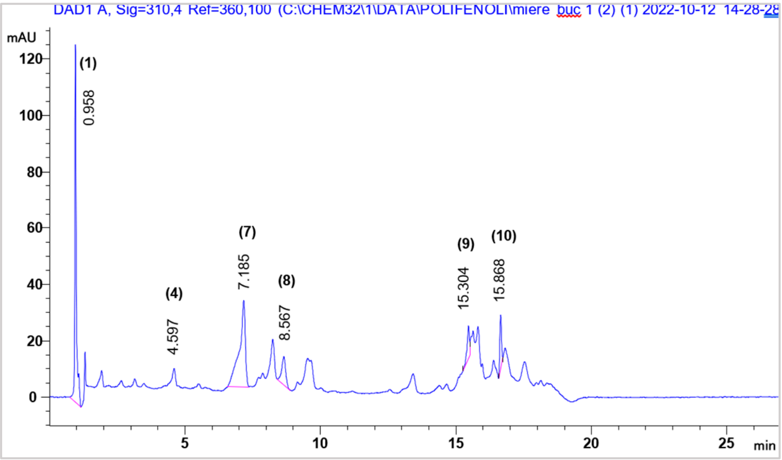

Figure S3. HPLC chromatographic profile of the hydroalcoholic extract of Black locust honey (Romania). The identified compounds: (1) gallic acid, (4) caffeic acid, (7) p-coumaric acid, (8) ferulic acid, (9) ellagic acid, (10) cinnamic acid

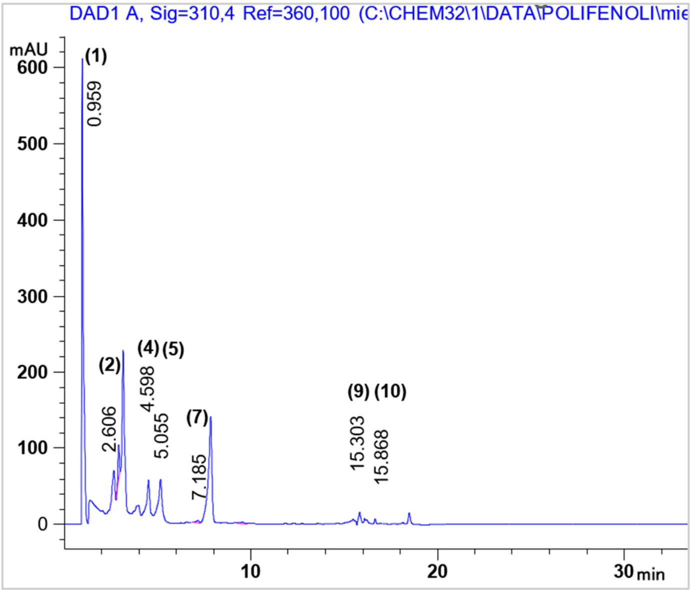

Figure S4. HPLC chromatographic profile of the hydroalcoholic extract of Multiflower honey (Romania). The identified compounds: (**1**) gallic acid, (**2**) 3-o-methylgallic acid; (**4**) caffeic acid, (**5**) syringic acid, (**9**) ellagic acid, (**10**) cinnamic acid.

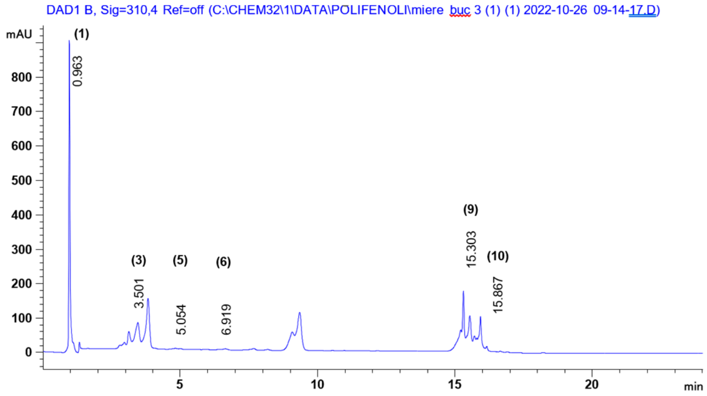

Figure S5. HPLC chromatographic profile of the hydroalcoholic extract of Chestnut honey (Spain). The identified compounds: (**1**) gallic acid, (**3**) chlorogenic acid, (**5**) syringic acid, (**6**) vanillin, (**9**) ellagic acid, (**10**) cinnamic acid.

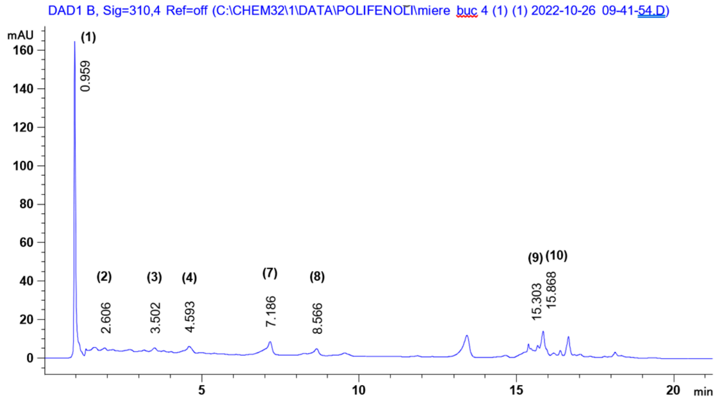

Figure S6. HPLC chromatographic profile of the hydroalcoholic extract of Thyme honey (Romania). The identified compounds: (1) gallic acid, (2) 3-o-methylgallic acid; (3) chlorogenic acid, (4) caffeic acid, (7) p-coumaric acid, (8) ferulic acid, (9) ellagic acid, (10) cinnamic acid.

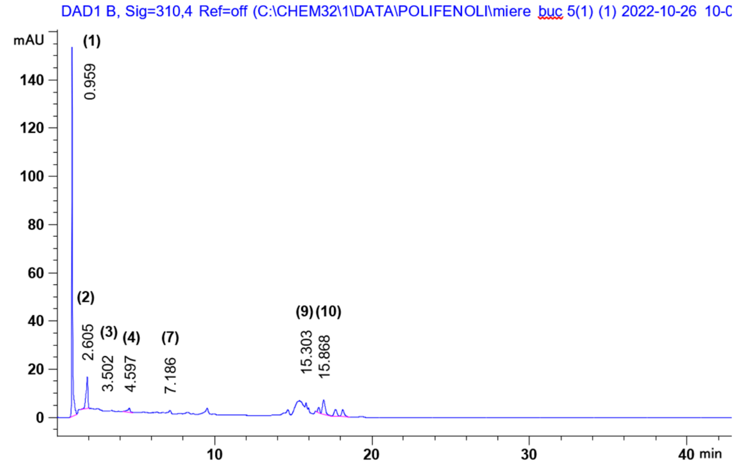

Figure S7. HPLC chromatographic profile of the hydroalcoholic extract of Pasture honey (Romania). The identified compounds: (1) gallic acid, (2) 3-o-methylgallic acid; (3) chlorogenic acid, (4) caffeic acid, (7) p-coumaric acid, (9) ellagic acid, (10) cinnamic acid.

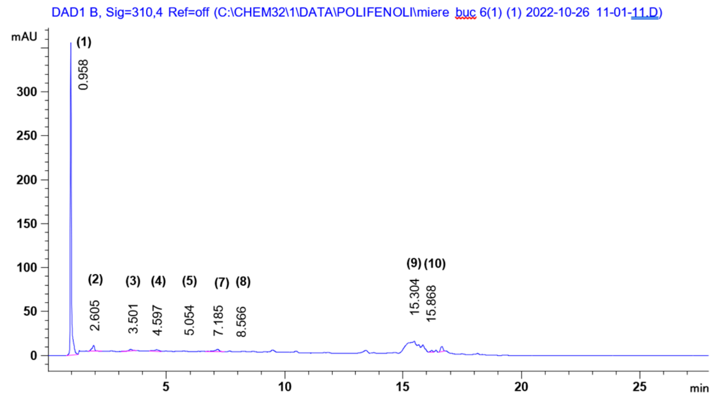

Figure S8. HPLC chromatographic profile of the hydroalcoholic extract of Manna honey (Romania). The identified compounds: (1) gallic acid, (2) 3-o-methylgallic acid; (3) chlorogenic acid, (4) caffeic acid, (5) syringic acid, (7) p-coumaric acid, (8) ferulic acid, (9) ellagic acid, (10) cinnamic acid.

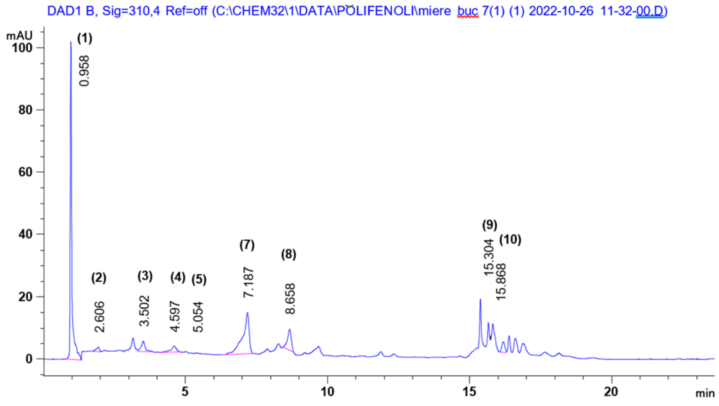

Figure S9. HPLC chromatographic profile of the hydroalcoholic extract of Mint honey (Romania). The identified compounds: (1) gallic acid, (2) 3-o-methylgallic acid; (3) chlorogenic acid, (4) caffeic acid, (5) syringic acid, (7) p-coumaric acid, (8) ferulic acid, (9) ellagic acid, (10) cinnamic acid.

.

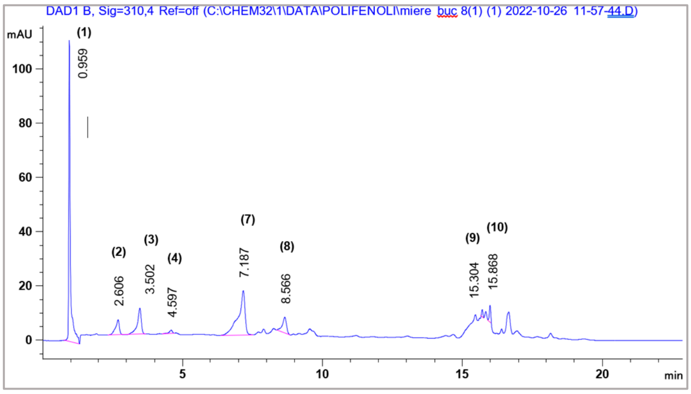

Figure S10. HPLC chromatographic profile of the hydroalcoholic extract of Hawthorn honey (Romania).The identified compounds: (1) gallic acid, (2) 3-o-methylgallic acid; (3) chlorogenic acid, (4) caffeic acid, (7) p-coumaric acid, (8) ferulic acid, (9) ellagic acid, (10) cinnamic acid.

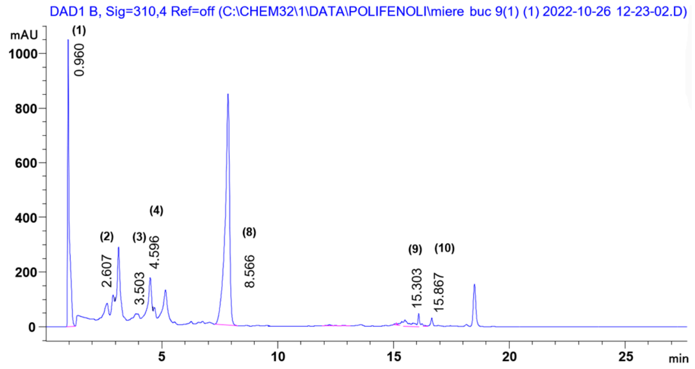

Figure S11. HPLC chromatographic profile of the hydroalcoholic extract of Rapeseed honey (Romania). The identified compounds: (1) gallic acid, (2) 3-o-methylgallic acid; (3) chlorogenic acid, (4) caffeic acid, (8) ferulic acid, (9) ellagic acid, (10) cinnamic acid.

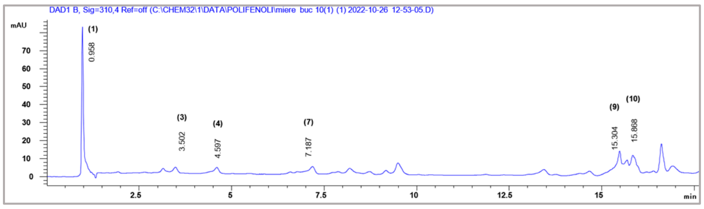

Figure S12. HPLC chromatographic profile of the hydroalcoholic extract of Linden honey (Romania). The identified compounds: (1) gallic acid; (3) chlorogenic acid, (4) caffeic acid, (7) p-coumaric acid, (9) ellagic acid, (10) cinnamic acid.

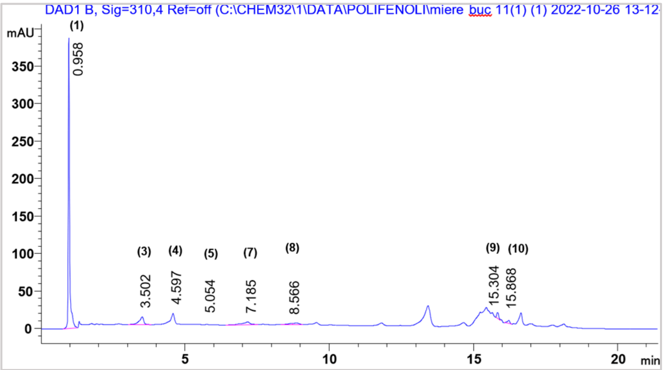

Figure S13. HPLC chromatographic profile of the hydroalcoholic extract of Manuka honey (New Zealand). The identified compounds: (1) gallic acid; (3) chlorogenic acid, (4) caffeic acid, (5) syringic acid, (8) ferulic acid, (9) ellagic acid, (10) cinnamic acid.

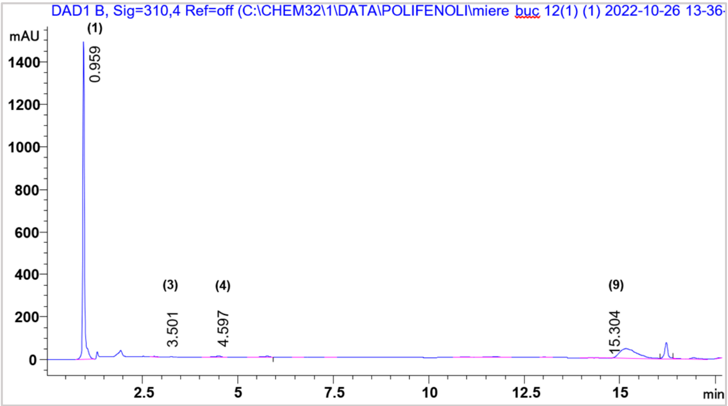

Figure S14. HPLC chromatographic profile of the hydroalcoholic extract of Tualang honey (Malaysia). The identified compounds: (1) gallic acid; (3) chlorogenic acid, (4) caffeic acid, (9) ellagic acid
